# Copper overload impairs hematopoietic stem and progenitor cell proliferation *via* prompting HSF1/SP1 aggregation and the subsequently downregulating *FOXM1-Cytoskeleton* axis

**DOI:** 10.1016/j.isci.2023.106406

**Published:** 2023-03-14

**Authors:** LingYa Li, ZhiPeng Tai, WenYe Liu, Yi Luo, You Wu, ShuHui Lin, Mugen Liu, BaoXiang Gao, Jing-Xia Liu

**Affiliations:** 1 College of Fisheries, Key Laboratory of Freshwater Animal Breeding, Ministry of Agriculture, Huazhong Agricultural University, Wuhan, Hubei 430070, China; 2 Key Laboratory of Molecular Biophysics of the Ministry of Education, College of Life Science and Technology, Huazhong University of Science and Technology, Wuhan, Hubei 430074, China; 3 Key Laboratory of Analytical Science and Technology of Hebei Province, College of Chemistry and Environmental Science, Hebei University, Baoding, Hebei 071002, China

**Keywords:** Molecular biology, Cell biology, Stem cells research

## Abstract

Unbalanced Cu homeostasis has been suggested to be associated with hematopoietic disease, but the roles of Cu overload in the hematopoietic system and the potential mechanisms are obscure. Here, we report a novel association and the novel potential pathways for Cu overload to induce proliferation defects in zebrafish embryonic hematopoietic stem and progenitor cells (HSPCs) *via* down-regulating expression of *foxm1*-*cytoskeleton* axis, which is conserved from fish to mammals. Mechanistically, we show the direct binding of Cu to transcriptional factors HSF1 and SP1 and that Cu overload induces the cytoplasmic aggregation of proteins HSF1 and SP1. These result in the reduced transcriptional activities of *HSF1* and *SP1* on their downstream *FOXM1* as well as the *FOXM1* transcriptional activities on cytoskeletons in HSPCs, which leads to ultimately cell proliferation impairment. These findings unveil the novel linkage of Cu overload with specific signaling transduction as well as the subsequent HSPC proliferation defects.

## Highlights

• Cu overload impairs HSPC proliferation
• *FOXM1* upstream of cytoskeletons in Cu overload-induced HSPC proliferationimpairment
• Cu directly binds HSF1/SP1 and affects the transcription of the *FOXM1*

## Graphical abstract

**Figure:**
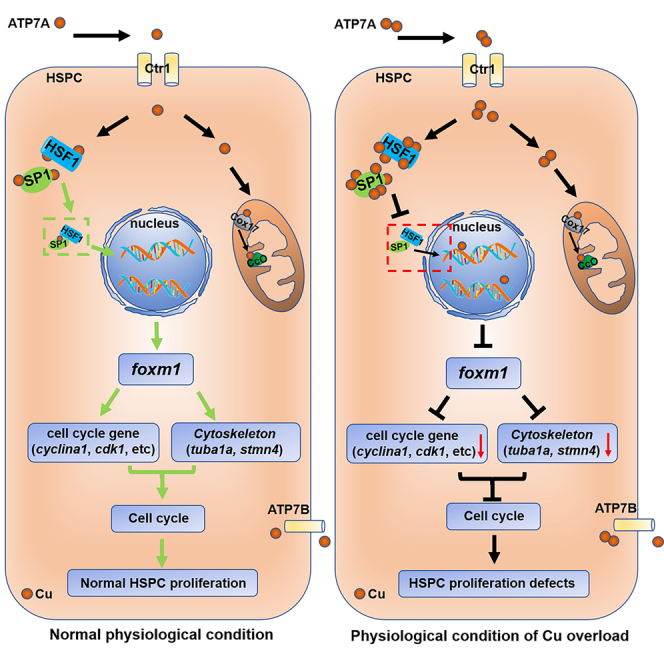

## Introduction

The unbalanced homeostasis of copper (Cu), an essential trace element in living organisms, can lead to developmental defects and various diseases. Cu deficiency causes the blockage of hematopoietic cell differentiation, leading to anemia, neutropenia and thrombocytopenia.^1^^,^^2^^,^^3^ Cu overload in the body can cause a variety of hematopoietic diseases, such as acute hemolytic anemia and leukemia,^4^ and higher serum Cu is observed in children with leukemia,^4^^,^^5^^,^^6^ suggesting unbalanced Cu homeostasis is strictly related to hematopoietic diseases although the potential differences and mechanisms are complicate and unknown.

The transcriptional and signaling orchestration is usually common in the development of both hematopoietic stem and progenitor cells (HSPCs) and leukemia, and the orchestration of the development of HSPCs is highly conserved from zebrafish to mammals.^7^ The HSPC emerge in zebrafish embryos initiates around 26-28 hours post fertilization (hpf) and is termed the definitive hematopoiesis, differentiating from *flk1* positive hemogenic endothelium located in aorta-gonad mesonephros (AGM), followed by migration to caudal hematopoietic tissue (CHT) and proliferation. The embryonic HSPCs can be transplantable,^8^ marked by genes *cmyb* and *runx1*.^9^ Recently, studies have unveiled that Cu overload induces embryonic skeletal myofibrillogenesis^10^ and neural system myelin defects,^11^ and impairs embryonic angiogenesis and lymphangiogenesis.^12^ Meanwhile, it is reported that Cu overload has a strong negative effect on zebrafish hematopoietic system *via* reducing hematopoiesis potential of head kidney in common carp,^13^ suggesting the potential linkage of Cu overload with hematopoiesis potential of HSPCs. In this study, we will study the roles of Cu overload in the emergence and proliferation of HSPCs.

A clear picture of the mechanisms underlying Cu overload-induced developmental defects during vertebrate embryogenesis has not yet emerged, although Cu overload impairs angiogenesis and lymphangiogenesis *via* blocking the migration rather than the proliferation of endothelial cells,^12^ induces myofibrillogenesis^10^ and myelination defects^11^ *via* epigenetic regulation, and induces retinal and intestinal development defects *via* triggering endoplasmic reticulum (ER) stress and reactive oxygen species (ROS) and the subsequent cell apoptosis.^14^ These findings suggest the differences of embryonic cells responding to Cu overload. Meanwhile, the cross-linkage of Cu and its binding molecules as regulators in cell cycle and proliferation^15^^,^^16^ suggest that Cu might target evolutionarily conserved cellular machinery in cell proliferation during embryonic development and tumor progress, however, such mechanisms have yet to be elucidated completely and thoroughly.

Cu homeostasis is accurately regulated by Cu transporters, and Cu exporters ATP7A and ATP7B^17^^,^^18^ are encoded by genes that are mutated in the Cu dysregulation syndromes Menkes disease and Wilson’s disease, respectively.^19^^,^^20^ Hematopoietic diseases such as hemolytic anemia is usually observed in Wilson’s disease,^21^^,^^22^ however, few reports the hematopoiesis potential in Wilson’s disease. Cu uptake is blocked in peripheral tissues such as in blood cells in Menkes disease for ATP7A mutants, and in the mutants Cu is failed to be pump to the circulation system from enterocytes.^22^^,^^23^ Meanwhile, Cu transporters COX17 participates in the delivery of Cu from the mitochondrial inter-membrane space to *cytochrome c oxidase* (CcO), the terminal enzyme in the mitochondrial energy-transduction respiratory chain, and studies have shown that Cu chaperone COX17 regulates the properties and differentiation of acute myeloid leukemia stem cells by controlling the level and distribution of mitochondrial Cu.^24^

In this study, we found that Cu overload at a certain concentration range could disrupt the cell cycle in both zebrafish HSPCs and mammalian cells, and zebrafish mutants of *cox17*^*−/−*^,^25^ *atp7a*^*−/−*^,^11^ and *atp7b*^*−/−*^^26^ were used as genetic models to unveil the naturally occurring disorders of Cu homeostasis in HSPC proliferation and to unveil the response of HSPC development to Cu stresses in different genetic mutants of Cu transporters. The purpose of our treatment of zebrafish embryos and mammalian cells with Cu was to address the following three questions: (1) which factors and signals mediate the Cu overload-induced zebrafish HSPC proliferation defects; (2) how Cu overload regulates the mediators and signals; (3) whether the function of Cu overload at a certain concentration range in the inhibiting cell cycle is highly conserved from fish to mammals.

## Results

### Cu overload in hematopoietic stem and progenitor cells and hematopoietic stem and progenitor cell development in copper stressed embryos

**Figure 1:**
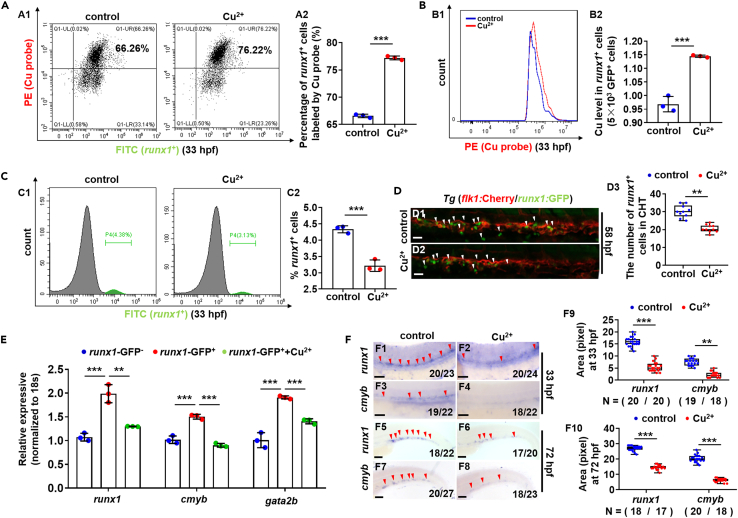
Accumulation of Cu in hematopoietic stem and progenitor cells (HSPCs) and the emergence and proliferation of HSPCs in Cu-stressed embryos (A and B) Proportion of HSPCs labeled with Cu probe (A) and the Cu content in individual *runx1*GFP^+^ cells (HSPCs) (B) were increased in Cu-stressed embryos at 33 hpf. A1, flow cytometry (FACS) plots; B1, flow cytometry (FACS) histogram; A2, calculation of the percentage of *runx1*GFP^+^ cells labeled by Cu probe; B2, calculation of Cu level in individual *runx1*GFP^+^ cells. (C) The percentage of *runx1*GFP^+^ cells in *runx1*:GFP embryos at 33 hpf. C1, flow cytometry (FACS) histogram; C2, calculation of the percentage of *runx1*GFP^+^ cells in different groups. (D) Cu-stressed *flk1:*Cherry*/runx1:*GFP embryos (D2) and the controls (D1) at 58 hpf. D3, calculation of *runx1*-positive cells (*runx1*^+^ cells). (E) The expression of HSPC genes *runx1*, *cmyb*, and *gata2b* in *runx1*GFP^−^ cells, *runx1*GFP^+^ cells, and Cu-stressed *runx1*GFP^+^ cells, respectively. (F) The expression of HSPC genes *runx1* and *cmyb* in embryos at 33 hpf and 72 hpf. F9, F10, calculation of *runx1* and *cmyb* expression level in the Cu-stressed and control embryos, respectively. Each experiment was repeated three times, and a representative result is shown. N_changed_/N_total_ in the right bottom corner of each panel indicates embryos with changed expression/total tested embryos, and N in calculation panels indicates the number of embryos with changed expression in each group. The same for the numbers in the following figures. F1-F8, lateral view, anterior to the left, and dorsal to the up. Data are mean ± SD. t-test, ∗p < 0.05, ∗∗p < 0.01, ∗∗∗p < 0.001. Scale bars, 20 μm (D1-D2), 50 μm (F1-F4), and 200 μm (F5-F8).

The collected *runx1* promoter driving GFP^+^ cells (*runx1*GFP^+^ cells) showed abundant expression of HSPC genes *runx1*, *cmyb*, and *gata2b*, while little expression of neural genes (*olig2*, *mbp*), liver gene (*fabp2*), and muscle gene (*myod*), suggesting the HSPC identity of the *runx1*GFP^+^ cells (Figures 1E and S1B) as reported recently.^27^ After FACS analysis of *runx1*GFP^+^ cells (HSPCs) labeled by Cu ion probe, the proportion of PE^+^GFP^+^ cells labeled with Cu probe (Figure 1A) and the Cu content in individual *runx1*GFP^+^ cells (HSPCs) (Figure 1B) were found to be significantly increased (p < 0.05) in the Cu-stressed embryos. Additionally, red fluorescence was observed in some cells but not in the *runx1*GFP^+^ cells of the whole embryos labeled with Cu ion probe in low magnification (Figure S1C). HSPCs showed a significant reduction in the number and percentage in Cu-stressed embryos at both 33 hpf (Figure 1C) and 58 hpf (Figure 1D), with significantly reduced expression of the HSPC genes *runx1*, *cmyb* (Figures 1E and 1F), and *gata2b* (Figure 1E), suggesting the intracellular Cu accumulation and the subsequently impaired the production of HSPCs.

Additionally, posterior lateral mesoderm gene *pax2a* and trunk mesoderm gene *myoD* exhibited no obviously changed expression in Cu-stressed zebrafish embryos at 14 hpf (Figures S2C1 and S2C2), and up-regulated expression of *scl* with no changed expression of *gata1a*, *mpx*, *fli1* and *flk1* existed in Cu-stressed zebrafish embryos (Figures S2C3–S3C12), suggesting Cu overload exerted little effects on primitive hematopoiesis in zebrafish embryos, and the intracellular Cu accumulation induced impaired production of HSPCs was specific.

### Effect of copper chelator tetrathimolybdate and copper ionophores elesclomol on hematopoietic stem and progenitor cell development in zebrafish embryos

Copper transporter 1 (Ctr1) is a membrane Cu transporter that plays essential roles in Cu acquisition and for zebrafish development.^28^ In this study, gene *ctr1* showed identical expression in *runx1*GFP^+^ cells and in *runx1*GFP^−^ cells, while Cu stresses significantly suppressed *ctr1* transcriptional expression in *runx1*GFP^+^ cells at 33 hpf (Figure S2B1) and in the whole embryos at 33 hpf and 72 hpf (Figures S2B2–S2B5).

**Figure 2:**
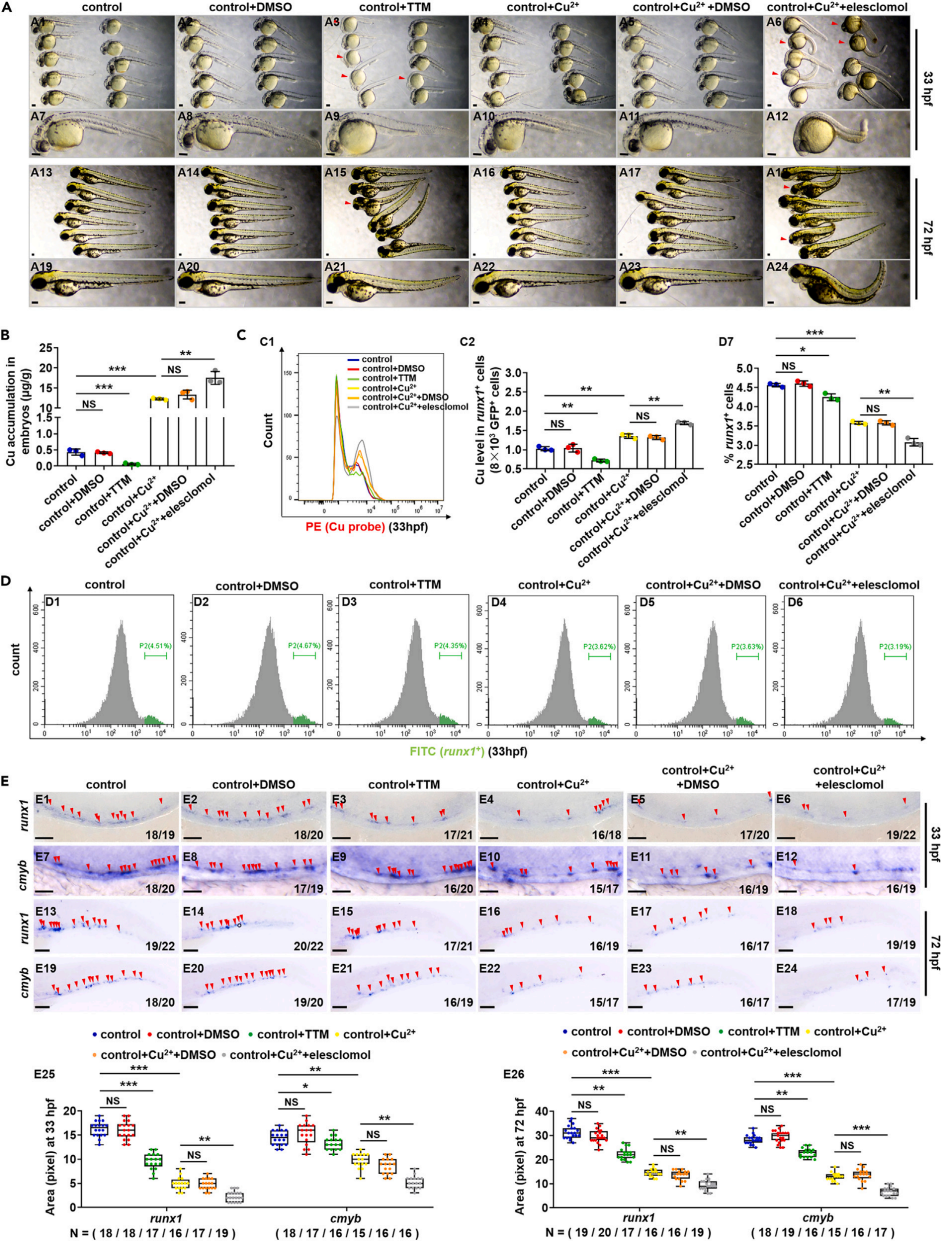
Cu chelator TTM and ionophore elesclomol affect embryonic and HSPC development during zebrafish embryogenesis (A) Phenotypes of zebrafish embryos in the control, DMSO exposed, TTM exposed, Cu exposed, Cu and DMSO co-exposed, or Cu and elesclomol co-exposed group, at 33 hpf and 72 hpf, respectively. (B) Cu concentration in zebrafish embryos from the control, DMSO exposed, TTM exposed, Cu exposed, Cu and DMSO co-exposed, or Cu and elesclomol co-exposed group at 33 hpf, respectively. (C) Cu content in individual *runx1*GFP^+^ cells (HSPCs) of zebrafish embryos in control, DMSO exposed, TTM exposed, Cu exposed, Cu and DMSO co-exposed, or Cu and elesclomol co-exposed group at 33 hpf, respectively. C1, flow cytometry (FACS) histogram; C2, calculation of Cu level in individual *runx1*GFP^+^ cells. (D) The percentage of *runx1*GFP^+^ cells in *runx1*:GFP embryos from different groups at 33 hpf, respectively. D1-D6, flow cytometry (FACS) histogram; D7, calculation of the percentage of *runx1*GFP^+^ cells in different groups. (E) The expression of HSPC genes *runx1* and *cmyb* in embryos from different groups at 33 hpf and 72 hpf. E25, E26, calculation of *runx1* and *cmyb* expression level in embryos from different groups, respectively. A1-A24, E1-E24, lateral view, anterior to the left, and dorsal to the up. Data are mean ± SD. t-test, ∗p < 0.05, ∗∗p < 0.01, ∗∗∗p < 0.001. NS, not significant. Scale bars, 50 μm (E1-E12), 100 μm (A1-A24) and 200 μm (E13-E24).

Cu chelators remove copper ions from the body, but Cu ionophores are small molecules binding copper ions and help transport copper ions into the cell.^29^^,^^30^ To further identify the roles of intracellular Cu accumulation in HSPC development, we treated the embryos with TTM and elesclomol respectively and tested their HSPC development. TTM-treated embryos exhibited hypo-pigmentation at 33 hpf (Figures 2A3 and 2A9), and the embryos exhibited a slightly developmental delay with shorten body and enlarged yolk sac at 72 hpf (Figures 2A15 and 2A21). Meanwhile, the embryos in Cu^2+^ and elesclomol co-treated group exhibited obvious trunk abnormalities, enlarged yolk sac, and thoracic enlargement at 33 hpf and 72 hpf (Figures 2A6, 2A12, 2A18, and 2A24).

Compared with the Cu concentration in the control embryos at 33 hpf, it was significantly decreased (p < 0.05) to 0.07 ± 0.02 μg/g in TTM-treated embryos, was significantly increased (p < 0.05) to 12.29 ± 0.23 μg/g in Cu-stressed embryos, and was further significantly increased (p < 0.05) to 17.53 ± 1.63 μg/g in Cu and elesclomol co-treated embryos (Figure 2B). Consistently, the variation tends of Cu concentration in individual *runx1*GFP^+^ cells (HSPCs) at different treatments were similar to that of the total Cu concentration in zebrafish embryos from different groups (Figure 2C). The percentage of HSPCs and expression of the HSPC genes showed a slight reduction in TTM-treated embryos at 33 hpf, and their expression decreased sharply in Cu and elesclomol co-treated embryos (Figures 2D and 2E). Meanwhile, in Cu and elesclomol co-treated embryos, more concentrated Cu and more reduced HSPCs were observed compared to that in Cu single-treated embryos (Figures 2D and 2E).

### Cu overload inhibits hematopoietic stem and progenitor cell proliferation in zebrafish embryos

**Figure 3:**
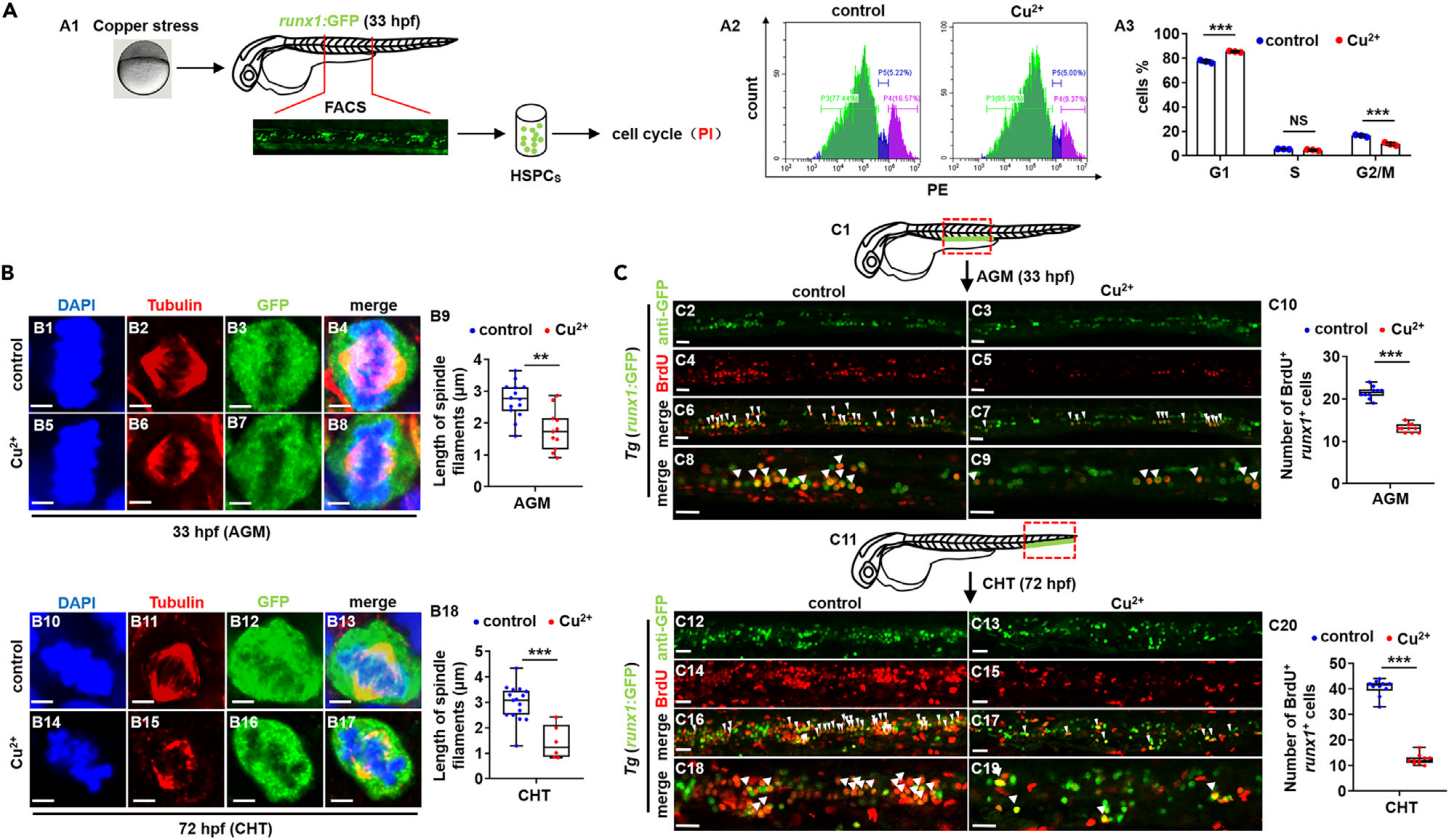
Cu overload induces HSPC proliferation impairment (A) Cell cycle of HSPCs in Cu-stressed embryos at 33 hpf. A1, Schema for the experiments; A2, flow cytometry (FACS) histogram; A3, cell cycle stage calculation. (B) Mitotic malformation of HSPCs in Cu-stressed embryos at 33 hpf (AGM) and 72 hpf (CHT), respectively. B1, B5, B10, B14, DAPI staining; B2, B6, B11, B15, anti-α-tubulin staining; B3, B7, B12, B16, anti-GFP staining; B4, B8, B13, B17, merged. At least 10 mitotic HSPCs in more than 10 embryos were observed for each group. B9, B18, calculation the length of spindles in metaphase *runx1*GFP^+^ cells at 33 hpf and 72 hpf, respectively. (C) BrdU cell proliferation assays in embryos at 33 hpf (C2-C9) and 72 hpf (C12-C19), respectively. C1, C11, AGM and CHT domain in embryos, respectively; C10, C20, calculation of the number of proliferative HSPCs in embryos from different groups. C2-C9, C12-C19, lateral view, anterior to the left, and dorsal to the up. Data are mean ± SD. t-test, ∗p < 0.05, ∗∗p < 0.01, ∗∗∗p < 0.001. NS, not significant. Scale bars, 2 μm (B1-B8, B10-B17) and 20 μm (C2-C9, C12-C19).

Cell cycle damage was potentially underlying mechanisms for reduced cell emergence during embryogenesis,^31^^,^^32^ so *runx1*GFP^+^ cells (HSPCs) were sorted at 33 hpf and used first for cell cycle detection in this study (Figure 3A1). The proportion of HSPCs was reduced significantly (p < 0.05) at G2/M stage from 16.57 ± 1.92% to 9.37 ± 2.63%, while increased (p < 0.05) at the G1 stage from 77.44 ± 2.45% to 85.38 ± 1.12% in Cu-stressed embryos at 33 hpf (Figures 3A2 and 3A3), with significantly reduced expressions of the genes in cell cycle in the Cu overload HSPCs (Figure S2D), suggesting Cu overload damaged cell cycle of HSPCs.

The spindle filaments of HSPCs in metaphase became shorter in Cu-stressed embryos at 33 hpf (Figures 3B1–3B8) and 72 hpf (Figures 3B10–3B17), and the spindle filaments in the other non-GFP^+^ cells also became disordered in the Cu-stressed embryos at 33 hpf (Figure S2E), suggesting Cu overload damaged chromosome alignment and segregation during mitosis. Furthermore, in Cu-stressed embryos, Anti-GFP^+^BrdU^*+*^ cells (HSPCs) were dramatically reduced in AGM (Figures 3C2–3C10) and CHT region (Figures 3C12–3C20), with reduced anti-phospho-histone 3 (PH3) positive staining in AGM and CHT region (Figure S2F), further strengthening a functional link between Cu overload and cell cycle of HSPCs.

### Cytoskeleton regulates copper overload-induced hematopoietic stem and progenitor cell deficiency

**Figure 4:**
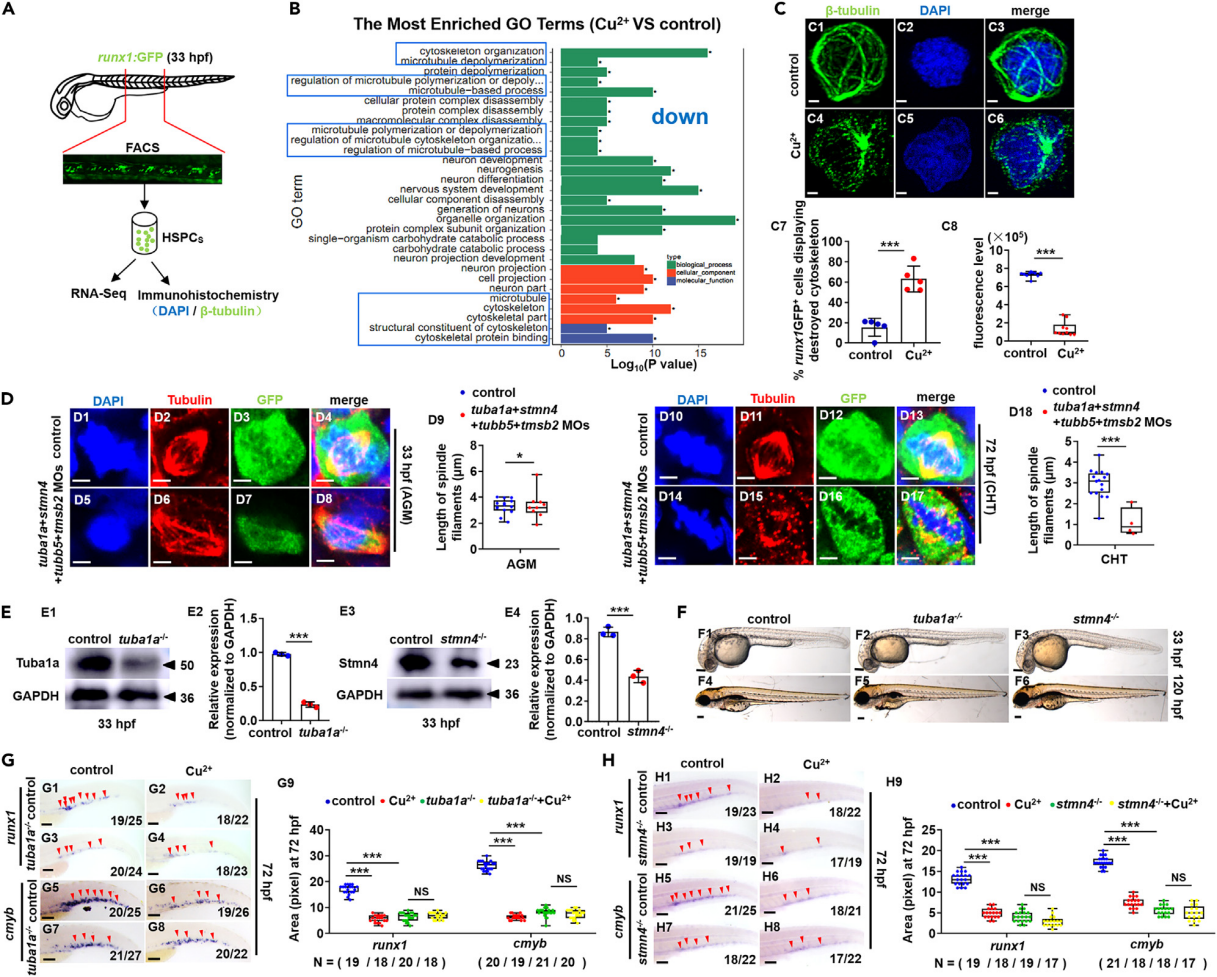
Cu overload destroys embryonic HSPC cytoskeleton (A) Schema for the experiments in (B) and (C). (B) Enriched GO terms for down-regulated DEGs in Cu-stressed HSPCs at 33 hpf. (C) Cytoskeleton protein immune-fluorescence for Cu-stressed HSPCs at 33 hpf. C1, C4, anti-β-tubulin staining; C2, C5, DAPI staining; C3, C6, merged; C7, the percentage of the Cu-stressed *runx1*GFP^+^ cells exhibiting destructive cytoskeleton; C8, calculation of the fluorescence intensity in each cell. (D) Mitotic malformation of HSPCs in the cytoskeleton gene morphants (*tuba1a*-MO, *stmn4*-MO, *tubb5*-MO, and *tmsb2*-MO together) at the 33 hpf (AGM) and 72 hpf (CHT), respectively. D1, D5, D10, D14, DAPI staining; D2, D6, D11, D15, anti-α-tubulin staining; D3, D7, D12, D16, anti-GFP staining; D4, D8, D13, D17, merged. At least 10 mitotic HSPCs in more than 10 embryos were observed for each group. D9, D18, calculation of the length of spindles in metaphase *runx1*GFP^+^ cells at 33 hpf and 72 hpf, respectively. (E) The protein level of Tuba1a (E1) and Stmn4 (E3) in *tuba1a* mutants and *stmn4* mutants at 33 hpf, respectively. GAPDH was used as an internal control. (F) Phenotypes of *tuba1a* (F2, F5) mutants with a 4-bp deletion and *stmn4* (F3, F6) mutants with an 8-bp deletion at 33 hpf and 120 hpf, respectively. (G) Expression of *runx1* and *cmyb* in *tuba1a* mutants with or without Cu stresses at 72 hpf. G9, calculation of *runx1* and *cmyb* expression in embryos from different groups. (H) Expression of *runx1* and *cmyb* in *stmn4* mutants with or without Cu stresses at 72 hpf. H9, calculation of *runx1* and *cmyb* expression in embryos from different groups. F1-F6, G1-G8, H1-H8, lateral view, anterior to the left, and dorsal to the up. Data are mean ± SD. t-test, ∗p < 0.05, ∗∗p < 0.01, ∗∗∗p < 0.001. NS, not significant. Scale bars, 1.2 μm (C1-C6), 2 μm (D1-D8, D10-D17), 100 μm (D1-D3, F1-F6), and 200 μm (G1-G8, H1-H8).

*Runx1*GFP^+^ cells (HSPCs) were sorted at 33 hpf for RNA deep sequencing (RNA-Seq) analysis (Figure 4A and Tables S11, S12, S13, S14, S15, S16, S17, and S18). RNA-Seq unveiled the elevated erythrocyte differentiation and myeloid cell homeostasis (Figures S3A3, S3A4 and Tables S11, and S12) as reported recently.^33^^,^^34^ Differentially expressed cytoskeleton genes exhibited significantly reduced expression and enrichment in Cu overload HSPCs (Figures 4B and S3A2), which was further confirmed by qRT-PCR (Figure S3B) and WISH assays (Figure S3C). Additionally, damage to the cytoskeleton of *runx1*GFP^+^ cells (HSPCs) and reduction in the cytoskeleton positive immunofluorescence signaling were observed (Figure 4C).

Four cytoskeletal genes (*tubala*, *stmn4*, *tmsb2*, *tubb5*) downregulated responding to Cu overload in HSPCs were selected for further analysis. The four morphants exhibited little developmental defects independent of *p53* (Co-injection of *p53*-MO is an effective strategy to test the specificity or off-target effects of MOs^35^^,^^36^^,^^37^ (Figure S4A). Individual gene knockdown further confirmed that impaired HSPC emergence and proliferation occurred in the four morphants independent of functional *p53* (Figures S4B–S4D). A striking HSPC-restricted phenotype that a remarkable reduction in *runx1*GFP^+^ cell (HSPCs) numbers (Figure S5B) and in G2/M stage HSPCs (Figure S5C), was found in the four cytoskeleton genes together morphants. Moreover, the cytoskeleton genes together morphants showed a similar spindle malformation of HSPCs in metaphase as that of HSPCs in Cu-stressed embryos (Figure 4D), with severely damaged cytoskeleton protein distribution and the down-regulated cytoskeleton positive fluorescence in HSPCs in embryos with knockdown of either cytoskeleton gene *tuba1a*, *stmn4*, *tmsb2*, or *tubb5* (Figure S5D). These findings are consistent with Cu’s distinctive connection to cytoskeleton-mediated HSPC cell cycle impairments.

Knockout studies confirmed that the deletion of *tuba1a* (4-bp deletion in exon 2) (Figures S6A, S6C1, S6C2, 4E1, and 4E2) and *stmn4* (8-bp deletion in exon 2) (Figures S6B, S6C3, S6C4, 4E3, and 4E4) impaired HSPC emergence and proliferation in embryos (Figures 4G, 4H, S6D, and S6E), although both the two mutants exhibited indistinguishable morphologies and no developmental delay (Figure 4F). Meanwhile, the injection of *tuba1a* mRNA or *stmn4* mRNA could rescue the expression of HSPC genes in *tuba1a*^−/−^ and *stmn4*^−/−^ mutants, respectively (Figures S6F and S6G), suggesting damaged cytoskeleton genes mediated Cu overload-induced HSPC defects.

### *Foxm1* acts upstream of cytoskeleton in mediating copper overload-induced hematopoietic stem and progenitor cell deficiency

**Figure 5:**
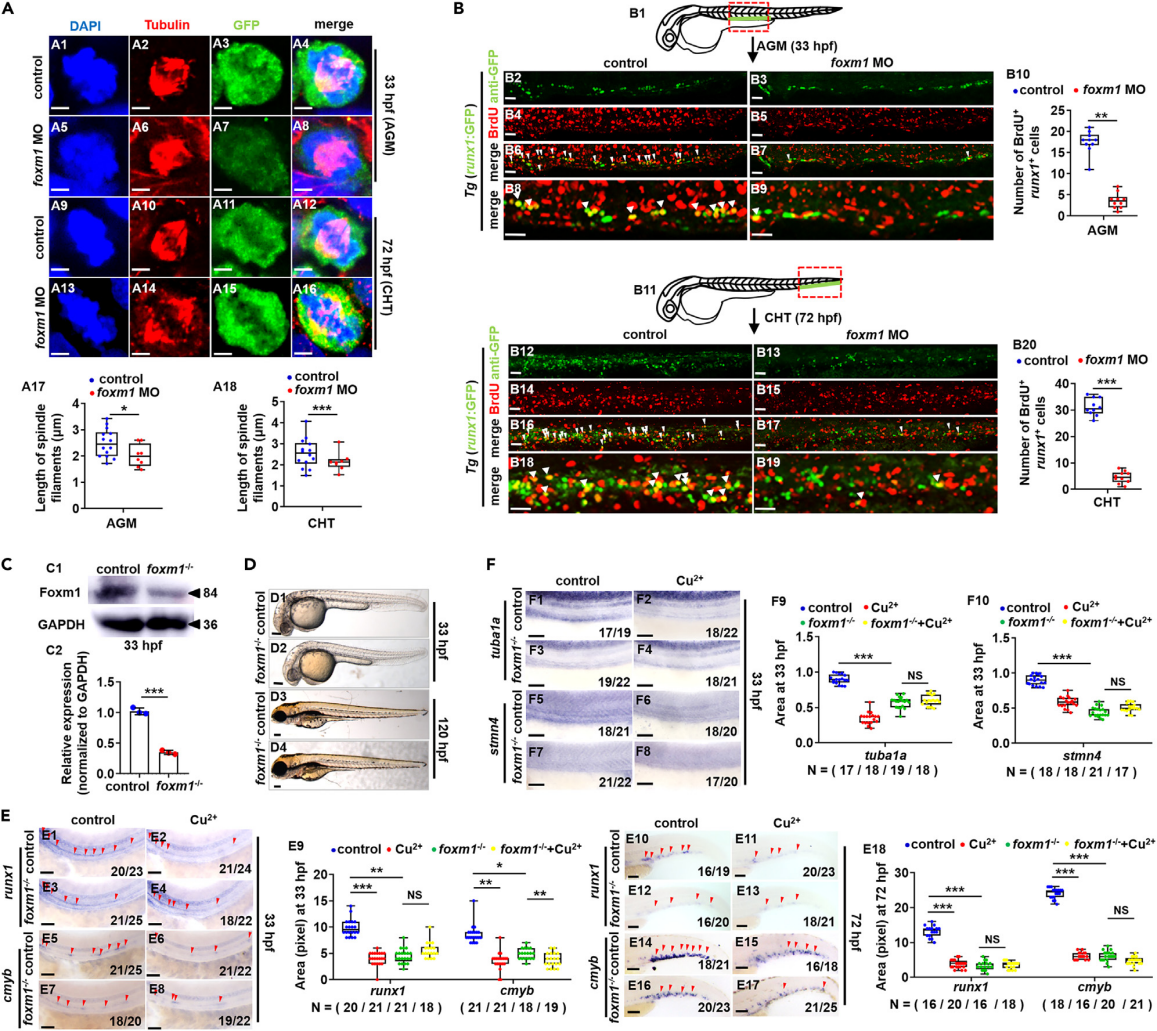
Dysfunction of gene *foxm1* impairs the proliferation of HSPCs (A) Mitotic malformation of HSPCs in *foxm1* morphants at 33 hpf (AGM) and 72 hpf (CHT), respectively. A1, A5, A9, A13, DAPI staining; A2, A6, A10, A14, anti-α-tubulin staining; A3, A7, A11, A15, anti-GFP staining; A4, A8, A12, A16, merged. At least 10 mitotic HSPCs in more than 10 embryos were observed for each group. A17, A18, calculation the length of spindles in metaphase *runx1*GFP^+^ cells at 33 hpf and 72 hpf, respectively. (B) BrdU cell proliferation in embryos at 33 hpf (B2-B9) and 72 hpf (B12-B19), respectively. B1, B11, AGM and CHT domain in embryos, respectively; B10, B20, calculation of HSPC proliferation in embryos from different groups. (C) The protein level of Foxm1 in *foxm1* mutants with a 10-bp deletion at 33 hpf. GAPDH was used as an internal control. (D) Phenotypes of *foxm1* mutants at 33 hpf and 120 hpf, respectively. (E) Expression of *runx1* and *cmyb* in *tuba1a* mutants with or without Cu stresses at 33 hpf and 72 hpf. E9, E18, calculation of *runx1* and *cmyb* expression in embryos from different groups. (F) Expression of *tuba1a* and *stmn4* in *foxm1* mutants with or without Cu stresses at 33 hpf. F9, F10, calculation of *tuba1a* and *stmn4* expression in embryos from different groups. B2-B9, B12-B19, D1-D4, E1-E8, E10-E17, F1-F8, lateral view, anterior to the left, and dorsal to the up. Data are mean ± SD. t-test, ∗p < 0.05, ∗∗p < 0.01, ∗∗∗p < 0.001, NS, not significant. Scale bars, 2 μm (A1-A16), 20 μm (B2-B9, B12-B19), 50 μm (E1-E8, F1-F8), 100 μm (D1-D4) and 200 μm (E10-E17).

We further checked the RNA-Seq data of both Cu overload embryos^38^ and HSPCs (this study) (Tables S19, S20, and S21), and found the down-regulated expression of *foxm1* (Tables S20 and S21) [which is essential for proper cell cycle progression by regulating the G1/S and G2/M transition and the execution of the mitotic program,^39^ is also required for maintaining HSPC pool in mammal adult^40^] (Figure S7A), and its reduced expression was further confirmed by qRT-PCR (Figure S7B1) and WISH (Figures S7B2–S7B4). Its knockdown induced a little developmental defect in zebrafish independent of gene *p53* (Figure S7C) as well as a significant reduction in the expression of HSPC markers (Figure S7D) and the number of *flk1*^+^*runx1*^+^ cells (Figure S7E). Additionally, *foxm1* knockdown led to a significant reduction (p < 0.05) of HSPCs at G2/M phase (Figure S7F), and showed disorganized spindle microtubule organization of HSPCs in metaphase (Figure 5A). The number of Anti-GFP^+^BrdU^*+*^ cells (HSPCs) in AGM (Figures 5B1–5B10) and CHT regions (Figures 5B11–5B20) was decreased significantly in *foxm1* morphants. Additionally, a *foxm1*-null mutant (10 bp deletion in the second exon) (Figures S8A, S8B, and 5C) exhibited almost normal-like phenotype (Figure 5D), but with the significant drop of HSPCs cells at both 33 hpf and 72 hpf (Figure 5E). Overexpression of *foxm1* rescued the HSPC phenotype in *foxm1*^−/−^ mutant (Figure S8C).

The down-regulated expression of *tuba1a*, *stmn4* and *tubb5* was observed in both *foxm1* morphants (Figure S7G) and mutants (Figures 5F1, 5F3, 5F5, 5F7, S8D1, and S8D3), similar to their expression tendency in Cu-stressed wild type embryos. Additionally, after Cu stimulation, *foxm1*^−/−^ mutants showed no obvious reduction in the expression of *tuba1a* (Figures 5F3 and 5F4), *stmn4* (Figures 5F7 and 5F8) or *tubb5* (Figures S8D3 and S8D4).

**Figure 6:**
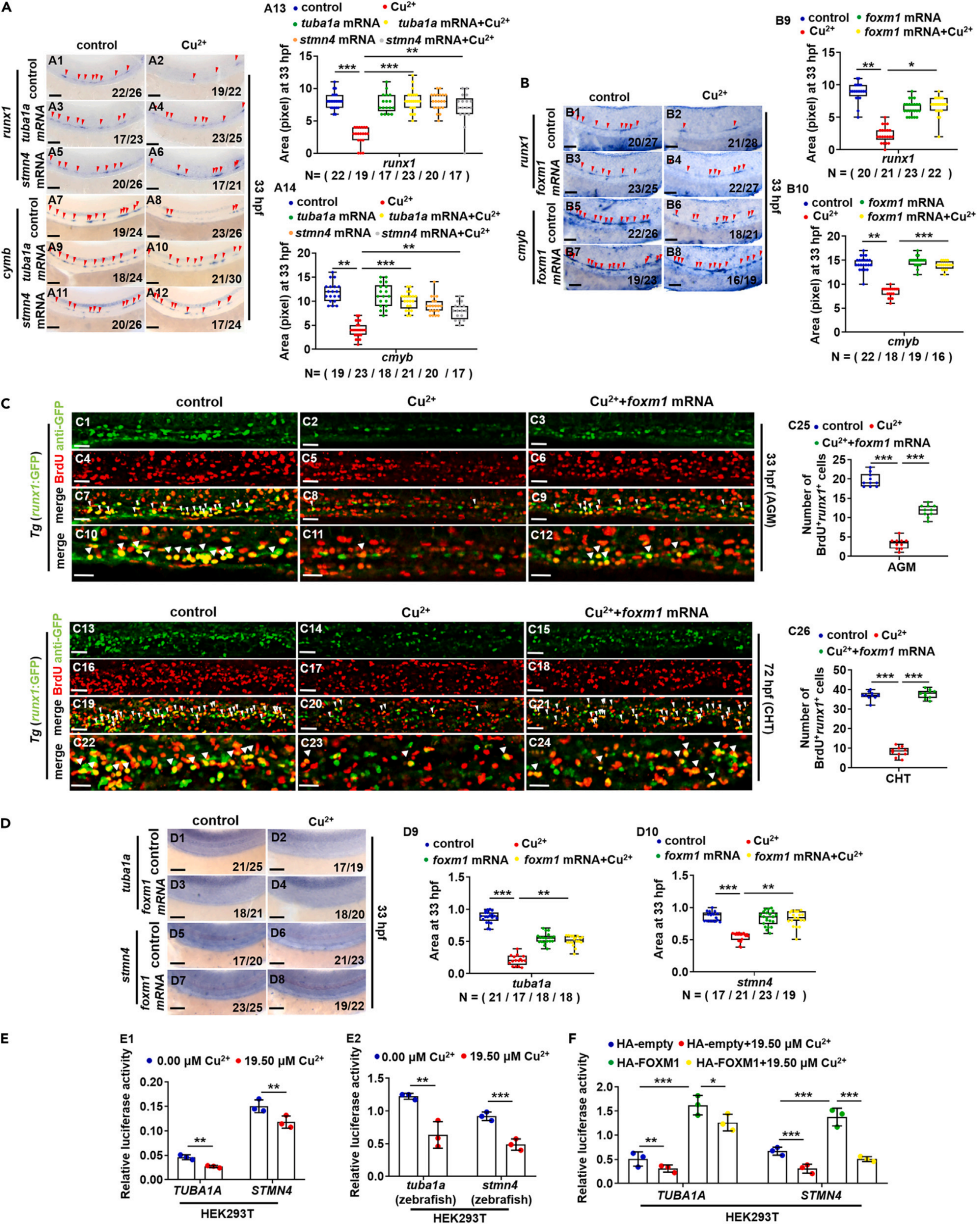
Ectopic expression of *tuba1a*, *stmn4,* or *foxm1* could recover the proliferation of HSPCs in Cu-stressed embryos (A) Ectopic expression of *tuba1a* and *stmn4* mRNA could recover the expression of *runx1* or *cmyb* in Cu-stressed embryos at 33 hpf. A13, A14, calculation of gene expression in each embryo from different groups. (B) Ectopic expression of *foxm1* mRNA could recover the expression of *runx1* or *cmyb* in Cu-stressed embryos at 33 hpf. B9, B10, calculation of gene expression in each embryo from different groups. (C) Ectopic expression of *foxm1* could recover BrdU cell proliferation in Cu-stressed embryos at 33 hpf (C1-C12) and 72 hpf (C13-C24). C25, C26, calculation of HSPC proliferation in embryos from different groups. (D) Ectopic expression of *foxm1* mRNA could recover the expression of *tuba1a* and *stmn4* in Cu-stressed embryos at 33 hpf. D9, D10, calculation of *tuba1a* and *stmn4* expression in embryos from different groups. (E) Cu significantly suppressed the transcriptional activities of both human (E1) and zebrafish (E2) *TUBA1A* and *STMN4* promoters. (F) Ectopic expression of human FOXM1 significantly up-regulated the transcriptional activities of *TUBA1A* and *STMN4* promoter and Cu overload significantly suppressed the up-regulated transcriptional activities. A1-A12, B1-B8, C1-C24, D1-D8, lateral view, anterior to the left, and dorsal to the up. Data are mean ± SD. t-test, ∗p < 0.05, ∗∗p < 0.01, ∗∗∗p < 0.001. Scale bars, 20 μm (C1-C24) and 50 μm (A1-A12, B1-B8, D1-D8).

The expressions of HSPC genes were recovered in Cu-stressed embryos *via* ectopic expression of *tuba1a*, *stmn4,* or *foxm1* mRNA separately (Figures 6A and 6B), and ectopic expression of *foxm1* could significantly recover GFP^+^BrdU^*+*^ cells (HSPCs) in AGM (Figures 6C1–6C12 and 6C25) and CHT regions (Figures 6C13–6C24 and 6C26) in Cu-stressed embryos. Meanwhile, ectopic expression of *foxm1* could significantly recover the expression of *tuba1a*, *stmn4* and *tubb5* in Cu-stressed embryos (Figures 6D and S8E). Cu could significantly suppress the transcriptional activities of both zebrafish and human *TUBA1A* and *STMN4* promoters (Figure 6E), which could be significantly up-regulated by ectopic expression of human *FOXM1*, but significantly down-regulated after Cu stresses (Figures 6F and S10A), strengthening a functional link between *FOXM1* and cytoskeleton machinery in Cu-induced HSPC cell cycle impairments, and *FOXM1* is located downstream of Cu but acts upstream of cytoskeleton genes.

### The roles of copper in impairing hematopoietic stem and progenitor cell proliferation are conserved in mammal cells

**Figure 7:**
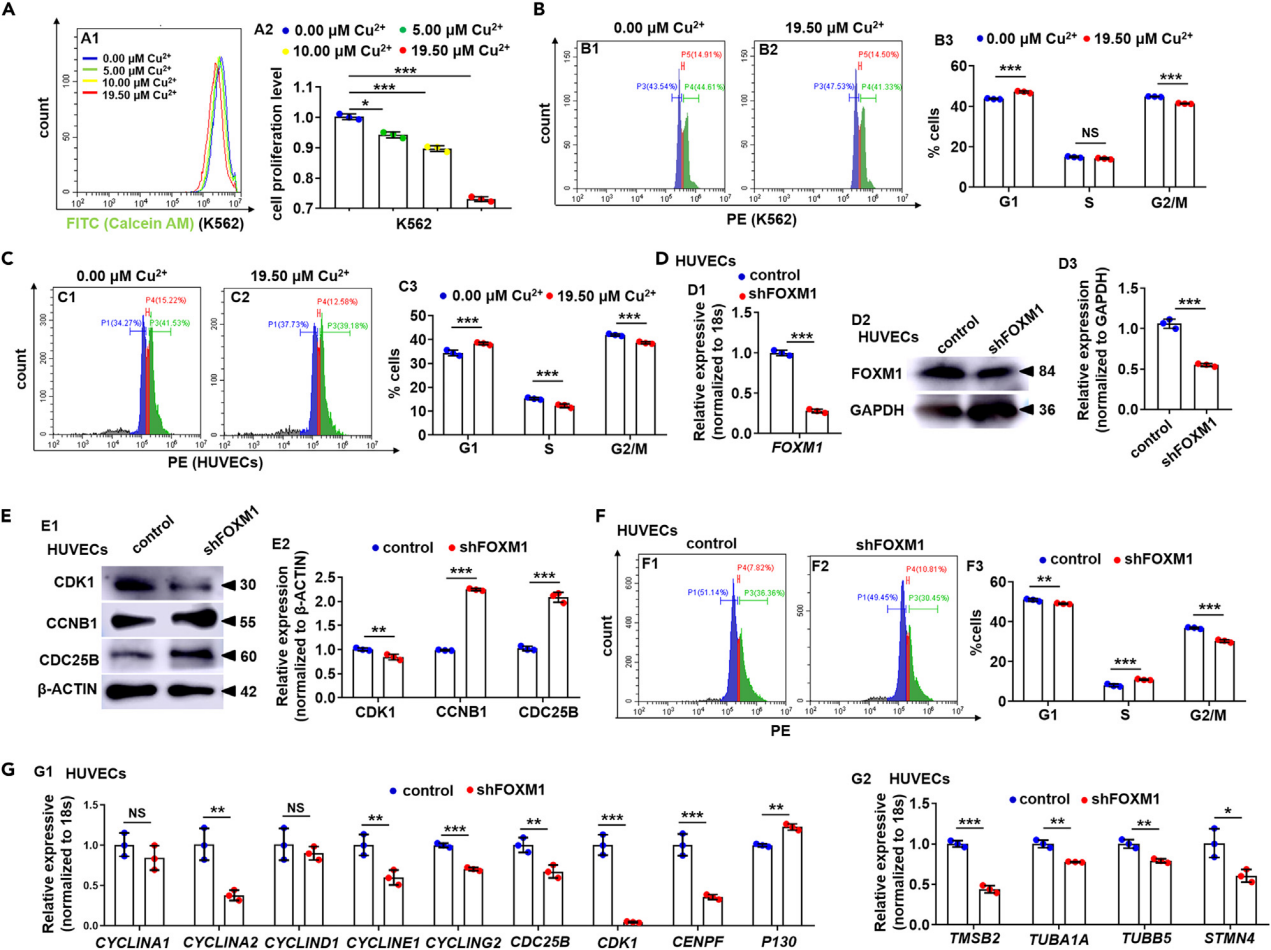
Cu overload impairs the cell cycle in mammalian cells (A) Analysis of cell proliferation in the 0.00 μM Cu, 5.00 μM Cu, 10.00 μM Cu, and 19.50 μM Cu-stressed K562 cells. A1, flow cytometry (FACS) histogram; A2, calculation of cell proliferation in different groups. (B) Cell cycle in 19.50 μM Cu-stressed K562 cells. B1, B2, flow cytometry (FACS) histogram; B3, calculation of cell cycle stage. (C) Cell cycle in 19.50 μM Cu-stressed HUVECs cells. C1, C2, flow cytometry (FACS) histogram; C3, calculation of cell cycle stage. (D) FOXM1 mRNA (D1) and the protein level (D2-D3) were decreased in *FOXM1* knockdown (shFOXM1) HUVECs, GAPDH was used as an internal control. (E) Protein levels of cell cycle regulators CDK1, CCNB1, and CDC25B in *FOXM1* knockdown (shFOXM1) HUVECs (E1), GAPDH was used as an internal control. (F) shFOXM1 HUVEC cells were blocked at the G1 stage. F1, F2, flow cytometry (FACS) histogram; F3, cell cycle stage calculation. (G) Expression of cell cycle genes (G1) and cytoskeleton genes (G2) in shFOXM1 cells. Data are mean ± SD. t-test, ∗p < 0.05, ∗∗p < 0.01, ∗∗∗p < 0.001. NS, not significant.

We further evaluated the cell proliferation and cell-cycle progression in mammal cells under Cu stresses. Cell proliferation level in the 5.00 μM Cu, 10.00 μM Cu, and 19.50 μM Cu-stressed K562 cells (The K562 line is established from pleural effusion during the blast crisis of a chronic myeloid leukemia patient,^41^ and is commonly used as an *in vitro* system to study HSPC biology^42^) were significantly reduced, to 94.23% ± 0.98%, 89.72% ± 0.94%, and 73.03% ± 0.80%, respectively (Figure 7A). A reduction (p < 0.05) of cells in the G2/M phase and a significant accumulation of cells in G1 was observed in K562 cells (Figure 7B), HUVECs (Figure 7C), and HEK293T (Figure S9A) cells after Cu stresses. Meanwhile, the expression of cell cycle-related genes (Figure S9B) and proteins CDK1, CDC25B, and CCNB1 (Figure S9C), and the expression of *FOXM1*, *TUBA1A*, and *TMSB2* were significant down-regulation (Figure S9D) in Cu-stressed HEK293T cells. Moreover, *FOXM1* knockdown decreased the percentage of cells in the G2/M phase as well as significantly down-regulated expression of cell cycle and cytoskeleton-related genes (Figures 7D-7G, S9E, and S9F).

### Copper directly binds transcriptional factors HSF1/SP1 and induces their cytoplasmic aggregation

**Figure 8:**
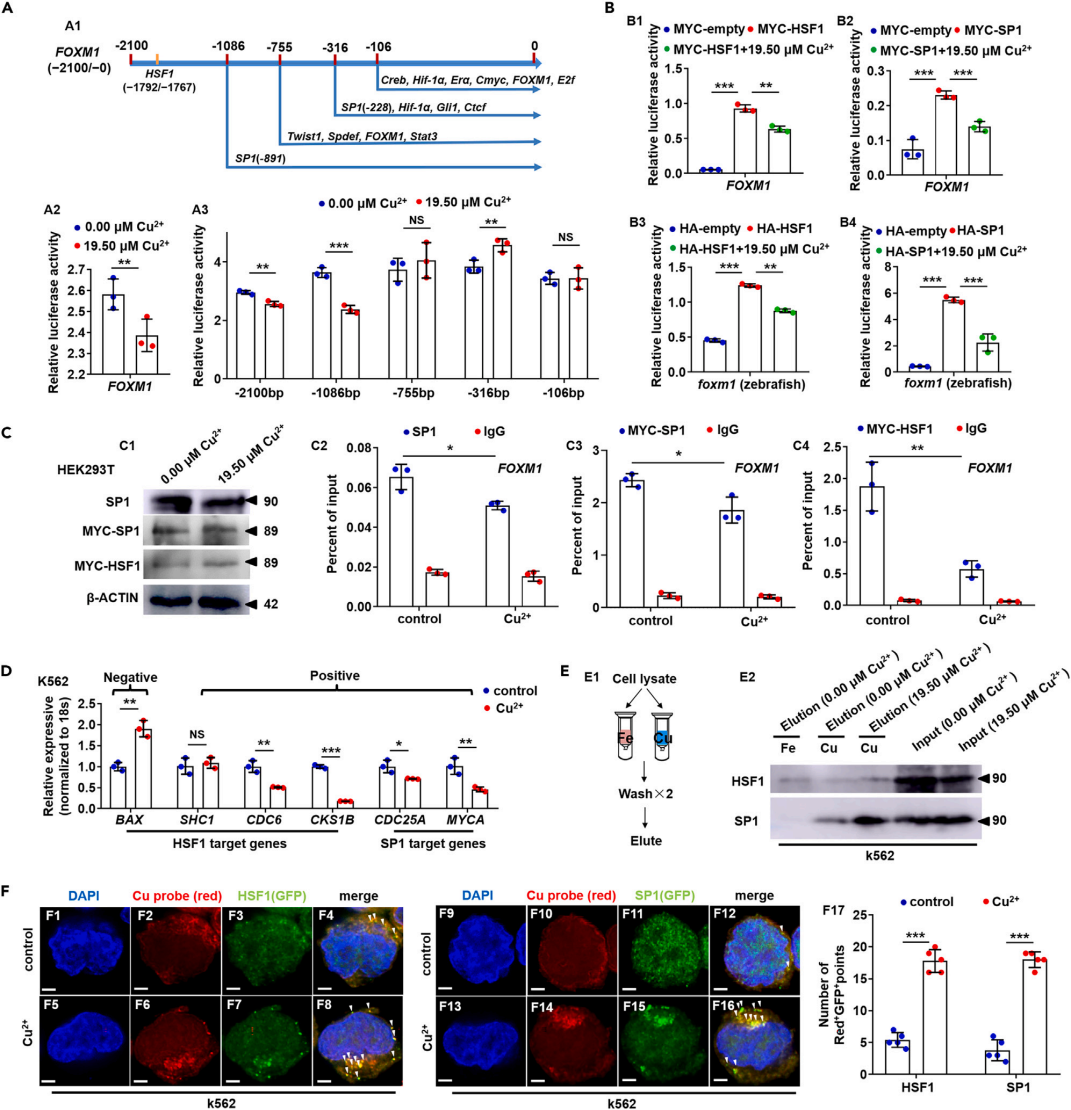
Cu overload impairs HSF1/SP1 transcriptional activities on *FOXM1* (A) Schematic diagram of transcription factor targeted domains in FOXM1 promoter (A1), Cu overload significantly suppressed the transcriptional activities of *FOXM1* promoters (A2) and transcriptional activities of the 5′ truncated promoters of gene *FOXM1* (−2100/+28, −1086/+28, −755/+28, −316/+28, −106/+28) (A3). (B) Ectopic expression of both zebrafish and human HSF1 or SP1 up-regulated the transcriptional activities of *FOXM1* promoter, and Cu overload inhibited the increased transcriptional activities on human (B1, B2) and zebrafish (B3, B4) *FOXM1* promoters, respectively. (C) Protein levels of SP1, MYC-SP1 (SP1), and MYC-HSF1 (HSF1) in Cu-stressed mammalian cells (C1). Reduced binding enrichment of protein SP1 (C2, C3) and HSF1 (C4) on FOXM1 promoter under Cu stresses as revealed by chromatin immunoprecipitation assays (ChIP). Anti-SP1 and Anti-MYC were used for ChIP assays in the control and the Cu-stressed cells, with anti-IgG used as the negative control. (D) Expression of HSF1 targeting genes BAX, SHC1, CDC6, CKS1B, and SP1 targeting genes CDC25A, MYCA in K562 cells. (E) The binding of the indicated proteins (HSF1/SP1) to Cu^2+^ and Fe^3+^ was assessed by western blot analysis of eluted proteins from the indicated metal-loaded resins. (F) Cu probe (Red^+^) and HSF1/SP1 protein (GFP^*+*^) double-positive foci were significantly increased in the cytoplasm of Cu overload K562 cells. F17, calculation of the number of Red^+^GFP^+^ foci in K562 cells from different groups. Data are mean ± SD. t-test, ∗p < 0.05, ∗∗p < 0.01, ∗∗∗p < 0.001. NS, not significant. Scale bars, 2 μm (F1-F16).

The transcriptional activities of human *FOXM1* promoter in cells were significantly decreased after Cu stresses (Figure 8A2). 5 truncated promoters of gene *FOXM1* (−2100/+28, −1086/+28, −755/+28, −316/+28, −106/+28) were constructed to unveil the Cu targeted domain on *FOXM1* promoter (Figure 8A1) based on previous report.^43^ Cu overload was shown to significantly suppress the transcriptional activities of −2100/+28 and −1086/+28 of *FOXM1* promoter rather than −755/+28, −316/+28, −106/+28 (Figure 8A3). HSF1 has been unveiled to effect on the −2100/-1086 region and SP1 on the −1086/-755 region on gene *FOXM1* promoter^43^ (Figure 8A1), and the transcriptional activities of *FOXM1* promoter were found to be significantly up-regulated by ectopic expression of both zebrafish and human HSF1 or SP1, but significantly inhibited after Cu stresses (Figures 8B and S10B). Meanwhile, ATOX1 is a Cu-dependent transcriptional factor in the activation of platelet-derived growth factor (PDGF), SOD3, and cyclinD1.^15^^,^^16^ In this study, ectopic expression of human ATOX1 inhibited the transcriptional activity of *FOXM1*, and its transcriptional activity was significantly inhibited after Cu stresses (Figures S10C1 and S10C3). However, ectopic expression of zebrafish ATOX1 did not change the transcriptional activity of *FOXM1* although its transcriptional activity was significantly inhibited after Cu stresses (Figures S10C2 and S10C4).

The protein levels remained unchanged in SP1 and HSF1 after Cu stresses (Figures 8C1 and S10D). The mRNA levels of *hsf1* and *sp1* showed identical in *runx1*GFP^+^ cells and in *runx1*GFP^−^ cells, and maintained stable in *runx1*GFP^+^ cells after Cu stresses (Figure S10E). However, SP1, MYC-SP1, or MYC-HSF1 ChIP-qPCR analyses showed that SP1 or HSF1 signals were less enriched at the promoter of *FOXM1* in Cu-stressed group compared with the control (Figures 8C2-8C4), and Cu overload significantly up-regulated expression of HSF1 negative target *BAX*^44^^,^^45^ while significantly down-regulated expressions of HSF1 positive target *CDC6* and *CKS1B*^46^ and SP1 positive targets *CDC25A*^47^ and *MYCA*^48^ (Figure 8D).

The experiments described above establish a connection between Cu and the expression of HSF1/SP1 but do not establish a direct mechanistic link. We hypothesized that Cu might directly bind to HSF1/SP1 proteins. To test this hypothesis, we purified HSF1/SP1 *via* Cu^2+^-charged resins from total K562 cell lysates as studies performed recently,^29^ and found that SP1 proteins bound to Cu^2+^-charged resin but not to Fe^3+^ resins, while HSF1 proteins bind to both Cu^2+^ and Fe^3+^ charged resins (Figures 8E and S10F). Meanwhile, Cu overload caused pronounced induction of Cu-HSF1/SP1 foci in the cytoplasm in K562 cells (Figure 8F), and Cu-SP1 foci were significantly more increased in K562 cells co-treated with Cu and elesclomol, but significantly decreased in K562 cells treated with TTM (Figure S10G).

### Mechanisms in copper overload-induced hematopoietic stem and progenitor cell proliferation is shared by genetic models with dysfunctional copper homeostasis

**Figure 9:**
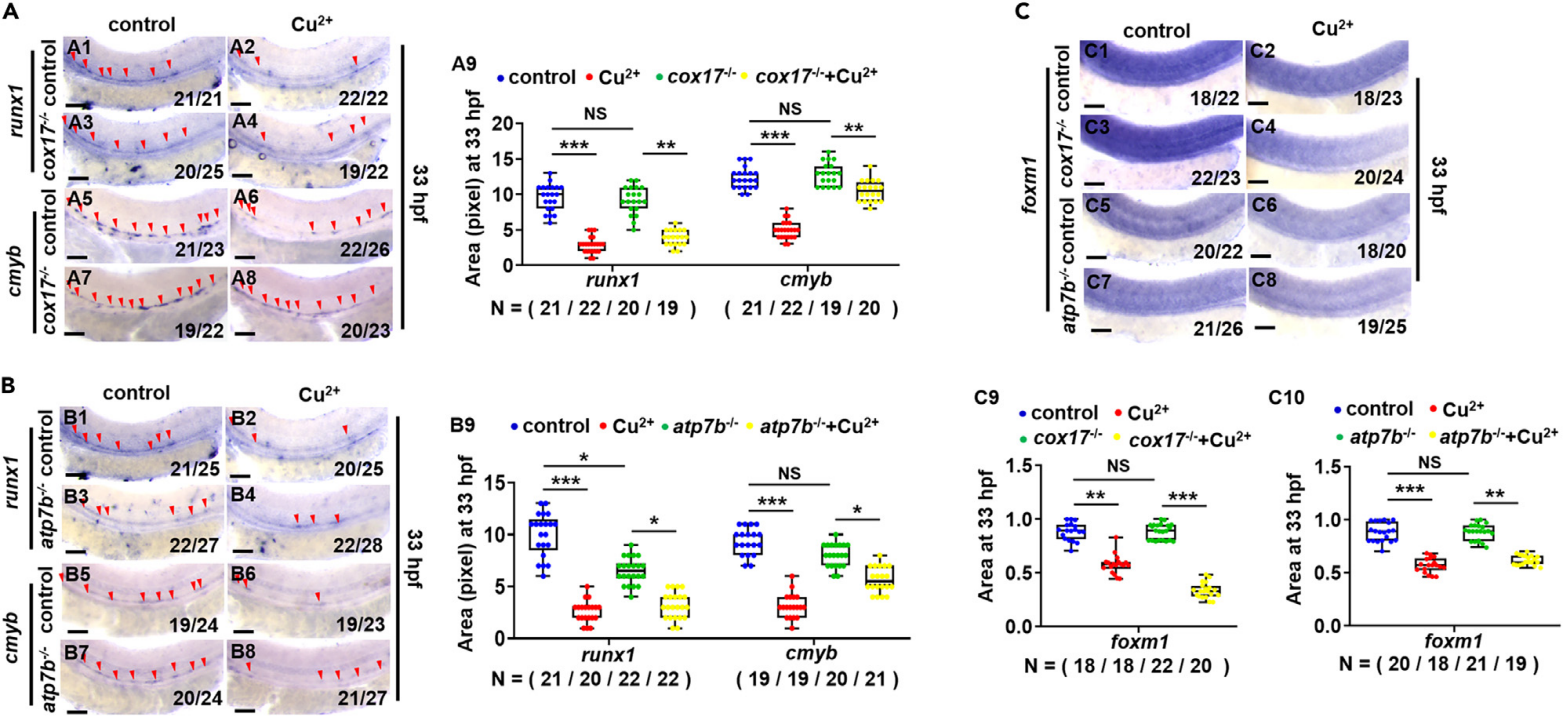
HSPC proliferation in genetic models with dysfunctional Cu homeostasis (A) Expression of *runx1* and *cmyb* in *cox17* mutants (*cox17*^*−/−*^) with or without Cu stresses at 33 hpf. A9, calculation of *runx1* and *cmyb* expression in embryos from different groups. (B) Expression of *runx1* and *cmyb* in *atp7b* mutants (*atp7b*^*−/−*^) with or without Cu stresses at 33 hpf. B9, calculation of *runx1* and *cmyb* expression in embryos from different groups. (C) Expression of *foxm1* in *cox17* mutants and *atp7b* mutants with or without Cu stresses at 33 hpf. C9, C10, calculation of *foxm1* expression in embryos from different groups. A1-A8, B1-B8, C1-C8, lateral view, anterior to the left, and dorsal to the up. Data are mean ± SD. t-test, ∗p < 0.05, ∗∗p < 0.01, ∗∗∗p < 0.001, NS, not significant. Scale bars, 50 μm (A1-A8, B1-B8, C1-C8).

To explore whether the Cu overload-induced HSPC developmental defects are shared by the naturally occurring disorders of Cu homeostasis in *cox17*-,^11^^,^^49^ *atp7b*-^11^ and *atp7a*-null mutants,^26^ and to test the roles of different Cu transports, *cox17*, *atp7b*, or *atp7a* in Cu overload-induced HSPC proliferation impairment, we tested the expression of *foxm1* and HSPC emergence and proliferation in the mutants with and without Cu stresses. No obviously changed expression of both *foxm1* and HSPC genes was observed in the three mutants (Figures 9A1, 9A3, 9A5, 9A7, 9B1, 9B3, 9B5, 9B7, 9C1, 9C3, 9C5, 9C7, S11A1, S11A3, S11A5, S11A7, S11D1, and S11D3), except that the expression of *runx1* was slightly down-regulated in the *atp7b*^−/−^ mutant (Figures 9B1 and 9B3). Meanwhile, after Cu stresses, expression of *runx1*, *cmyb*, and *foxm1* exhibited reduced expression in both *cox17*^−/−^ and *atp7b*^−/−^ mutants (Figures 9A3, 9A4, 9A7, 9A8, 9B3, 9B4, 9B7, 9B8, 9C3, 9C4, 9C7, and 9C8) rather than in *atp7a*^−/−^ mutants (Figures S11A3, S11A4, S11A7, S11A8, and S11B3, and S11B4).

## Discussion

It is well-known that the unbalanced Cu homeostasis in individuals is associated with hematopoietic system diseases even leukemia,^4^ and Cu ionophores (increasing bioavailable Cu, inducing Cu overload) have been suggested as agents in treating cancer which characterized with active cell proliferation,^50^^,^^51^ but little is known about the related molecular characteristics and underlying mechanisms. Here, we unveiled for the first time that bioavailable Cu at a certain concentration range could impair the cell cycle in both zebrafish HSPCs and mammalian cells, and show the effects of intracellular Cu on the development of zebrafish HSPCs. Overload Cu directly interacts with HSF1/SP1 and induces the cytoplasmic aggregation of the proteins. This results in the reduced transcriptional activities of the two proteins on *FOXM1* as well as FOXM1 transcriptional activities on cytoskeleton genes, which leads to the ultimately HSPC cell cycle and proliferation impairments.

Increased Cu content in HSPCs with a significant reduction in the number of nascent and fetal HSPCs as well as G2/M phase BrdU^+^ are observed in Cu overload embryonic HSPCs, strengthening that Cu induces HSPC developmental defects is primarily dependent on intracellular Cu accumulation. Moreover, we observe down-regulated expression of cytoskeleton genes and proteins as well as impaired cytoskeleton protein distribution in Cu overload HSPCs, which is consistent with reports that orderly and accurate rearrangement of cytoskeleton helps push the cell cycle forward,^52^^,^^53^ while their integrity disruptions lead to cell-cycle arrest.^52^^,^^54^ In this study, we demonstrate that Cu at a certain concentration range (nearly 2-fold increase) damages cell cycle, suggesting Cu might inhibit cell proliferation *via* inhibiting cell cycle, at the concentration which also induces cell senescence in zebrafish embryonic blood vessels^12^ and in mammalian cells.^55^

Meanwhile, the down-regulated expression of *foxm1* in Cu overload HSPCs, the spatial distribution disorder of cytoskeleton in HSPCs caused by *foxm1* knockdown/knockout, with the observation that the conservative roles of *FOXM1* in directly regulating cytoskeleton genes and cell cycle from fish to mammalian cells are shown in this study, consistently with reports that *FOXM1* regulate the cell cycle by regulating the structure of chromosomal bodies,^56^ activates the transcription of *CDC25B* requiring for G2/M transition,^57^^,^^58^ and affects both G1/S and G2/M transition during cell cycle.^57^ Consistently, this study observes the significantly reduced G2/M stage in the cell cycle in both Cu overload and in *FOXM1* knockdown embryonic HSPCs or mammalian cells and the significantly down-regulated expressions of CDK1 and CDC25B in Cu overload cells. Meanwhile, significantly down-regulated expression of CDK1 but increased expression of CDC25B is observed in *FOXM1* knockdown cells, indicating that cyclinA^59^ or EIF2α^60^ rather than CDC25B^61^ might regulate the expression of CDK1 in *FOXM1* knockdown cells.

Cu ionophores induced Cu overload is reported to reduce tumor growth and possess anticancer activities,^62^^,^^63^^,^^64^ and induce a distinct form of regulated cell death termed cuproptosis in a 15- to 60--fold increase intracellularly.^29^ Meanwhile, some studies have reported that Cu chelators (reducing bioavailable Cu) functioned effectively in the expansion or maintenance of progenitor cells *in vitro*^65^ as well as in patients with lung cancer (a high serum Cu level, >23.6 μM)^66^ and with breast cancers (average: 51.2 μM),^67^ suggesting Cu bioavailable or not might be the potential differences of system Cu overload in cell proliferation, in cell senescence, or in cell cuproptosis.^50^^,^^51^ In this study, we observe that Cu ionophore elesclomal co-treated with Cu induces more Cu probe positive fluorescence and more accumulated Cu in HSPCs and the resulted in more reduced percentage of HSPCs in embryos compared with that in Cu single-treated embryos. The observations in this study are not only consistent with reports that Cu ionophores induced Cu overload possess anticancer (anti-cell proliferation) activities, but also suggest that Cu overload impairs HSPC development is concentration-dependent. However, Cu chelator TTM not only induces reduced Cu probe positive fluorescence and little accumulated Cu in HSPCs compared with that in WT, but also lead to reduced percentage of HSPCs in embryos, suggesting Cu homeostasis is strictly for the emergence, maintenance, or amplification of HSPCs, Cu overload or Cu deficiency, both induce reduced HSPCs during zebrafish embryogenesis.

Cu significantly suppressed the transcriptional activities of *FOXM1* promoter in both −2100 to +28 and −1086 to +28 rather than in −775 to +28, −316 to +28, and −106 to +28, where transcriptional factors SP1(-891), HSF1(-1792/-1767), and so forth are pivotal in regulating *FOXM1* transcription.^43^ Consistently, we show that the ectopic expression of either *SP1* or *HSF1* could significantly up-regulate the *FOXM1* transcriptional activities, while the up-regulated transcriptional activities of both *SP1* and *HSF1* could be significantly inhibited by Cu overload. In this study, we unveil that Cu binds directly with transcriptional factors SP1 or HSF1, and Cu overload significantly reduces the binding enrichment of either SP1 or HSF1 in the gene *FOXM1* promoter, and induces the aggregation of proteins SP1 and HSF1 in the cytoplasm in HSPCs. These findings support a model that the down-regulated expression of *FOXM1* in HSPCs is mediated at least in part by the aberrant oligomerization of both SP1 and HSF1 proteins in the cytoplasm and their subsequently reduced functional transcriptional activities on *FXOM1* and other targeting genes *BAX*, *CDC6*, *CKS1B*, *CDC25A*, and *MYCA*. Consistently, a recent study also reports that Cu-induced cell death is mediated at least in part by the aberrant oligomerization of lipoylated TCA cycle proteins.^29^ ATOX1 is a well-known Cu-dependent transcriptional factor, however, this study unveils that Cu regulates *FOXM1* transcription independent of ATOX1. Additionally, different from ATOX1 dependent on Cu in the activation of PDGF, SOD3, and cyclin D1,^15^ this study unveils that Cu suppressed transcriptional activities of SP1 or HSF1 on gene *FOXM1*, suggesting the double-sided effects of Cu on nuclear transcriptional proteins depending on the Cu spatial distribution. Moreover, whether other Cu binding transcriptional factors in nuclear, such as MTF1/Nrf2, MT1/2, and ect.,^22^ are involved in Cu overload-induced HSPC proliferation defects, needs to be studied in the further days.

*HSF1* has been unveiled to be a master regulator in the heat shock response^68^^,^^69^ and in responses to environmental conditions such as metals and so forth ^70^ promotes hematopoietic stem cell fitness and proteostasis in response to *ex vivo* culture stress,^71^ activates beige fat metabolism against obesity.^72^ SP1, which belongs to the SP-like family, is a transcription factor that controls diverse cell functions and behaviors,^73^ and *SP1* has been shown to be required for the transcriptional regulation of Cu transporter *hCtr1* in response to the cellular Cu level^74^ and to be a pivotal transcriptional factor in early embryonic development.^75^ In this study, we show that Cu directly interacts with HSF1 and SP1 proteins and induces the cytoplasmic aggregation of the two transcriptional proteins. These findings expand the novel roles of both SP1 and HSF1 in linkage with Cu in the development of HSPCs, and widen the Cu binding transcriptional factors from MTF1/Nrf2, ATOX1, and ect., to MTF1/Nrf2, ATOX1, SP1, and HSF1, and ect..

In the case of genetic disorders of Cu homeostasis (*atp7b*^*−/−*^ for Wilson’s disease, *atp7a*^*−/−*^ for Menke’s disease, *cox17*^−/−^), our observations that no obvious change is observed in the expression of HSPC genes and *foxm1* in *atp7a*^−/−^ mutants with or without Cu stresses, and reduced expression of HSPC genes in *atp7b*^−/−^ and both *foxm1* and HSPC genes still exhibits down-regulated after Cu stresses in the mutants, not only strengthening the down-regulated expression of *foxm1* and HSPC proliferation impairment are primarily dependent on intracellular Cu accumulation because *atp7a*^−/−^ mutants lack the ability to pump Cu into the portal circulation and exhibit Cu overload in intestines while deficiency in other tissues,^76^ while *atp7b*^−/−^ mutants lack the ability to excrete excess cellular Cu,^77^ but also indicating hematopoiesis potential defects might be another contributor for anemia occurred in Wilson patients. Meanwhile, expression of gene HSPC genes and *foxm1* in *cox17*^−/−^ mutants do not change obviously before Cu stresses but still exhibits down-regulated after Cu stresses, excluding the effects of *cox17*-deletion-induced genetic disorders of Cu homeostasis on Cu overload-induced HSPC proliferation impairment.

In summary, this study shows that intracellular Cu overload effects negatively on the proliferation of zebrafish HSPCs, and confirms that Cu directly interacts with SP1 and HSF1 transcriptional proteins and Cu overload induces the aggregation of the two proteins in HSPCs, which leads to the reduced *SP1* and *HSF1* transcriptional activities on gene *FOXM1* and the down-regulation of cytoskeleton genes and the ultimately HSPC proliferation impairment. All the integrated data from this study might enrich the theoretical basis for the research of embryonic HSPC/blood cancer cell proliferation in a certain concentration range of trace element Cu, and provide some novel hints for the underlying mechanism of hematopoietic diseases in humans with unbalanced Cu homeostasis.

### Limitations of the study

In this study, we show that excess Cu directly binds transcriptional factors HSF1/SP1 and induces their cytoplasmic aggregation in the cytoplasm in cells, and whether it is a general effect for excess Cu on transcriptional factors in metal signaling transduction, we need more solid data to convince it in the future days.

## STAR★Methods

### Key resources table

**Table:** 

REAGENT or RESOURCE	SOURCE	IDENTIFIER
**Antibodies**		

Rabbit polyclonal anti-TUBA1A	Affinity Biosciences	Cat#AF7010; RRID: AB_2839418
Rabbit polyclonal anti-STMN4	Affinity Biosciences	Cat#DF4547; RRID: AB_2836898
Rabbit polyclonal anti-FOXM1	Affinity Biosciences	Cat#AF7860; RRID: AB_2844224
Rabbit monoclonal anti-CDK1	ABclonal	Cat#A11420; RRID: AB_2861564
Rabbit monoclonal anti-Cyclin B1	ABclonal	Cat#A19037; RRID: AB_2862529
Rabbit monoclonal anti-CDC25B	ABclonal	Cat# A9758; RRID: AB_2863774
Rabbit polyclonal anti-HSF1	ABclonal	Cat# A13765; RRID: AB_2760623
Rabbit polyclonal anti-HSF1	Abcam	Cat# ab52757, RRID: AB_880518
Rabbit polyclonal anti-SP1	ABclonal	Cat#A14662, RRID: AB_2761538
Rabbit monoclonal anti-Myc-Tag	ABclonal	Cat#AE070, RRID: AB_2863795
Rabbit monoclonal anti-HA-Tag	Covance	Cat#MMS-101P1000, RRID: AB_2770404
Rabbit monoclonal anti-beta (β-) Tubulin	ABclonal	Cat#A12289, RRID: AB_2861647
Mouse monoclonal anti-alpha (α-) Tubulin	GeneTex	Cat#GT114, RRID: AB_2716636
Rabbit monoclonal anti-beta (β-) Actin	ABclonal	Cat#AC026, RRID: AB_2768234
Rabbit polyclonal anti-GAPDH	ABclonal	Cat#AC001, RRID: AB_2619673
Mouse monoclonal anti-BrdU	ABclonal	Cat#A1482, RRID: AB_2756438
ABflo® 555-conjugated Goat Anti-Mouse IgG (H+L) secondary antibody	ABclonal	Cat#AS057, RRID: AB_2768321
Goat Anti-Rabbit IgG FITC (H+L) secondary antibody	biosharp	Cat#BL033A, RRID: AB_2769478
Goat Anti-Rabbit lgG (H+L)	biosharp	Cat#BL033A, RRID: AB_2769854
Goat Anti-Mouse IgG (H+L)	biosharp	Cat#BL001A, RRID: AB_2769851
Rabbit monoclonal anti-Phospho-Histone H3 (Ser10)	Cell Signaling Technology	Cat#53348, RRID: AB_2799431

**Bacterial and virus strains**		

DH5a competent *E. coli*	This paper	N/A

**Chemicals, peptides, and recombinant proteins**		

CuSO4·5H2O	Sigma	Cat#61245
Ammonium ferric citrate (Fe^3+^)	Macklin	Cat#C13259398
Elesclomol	Selleck	Cat#S1052
Tetrathiomolybdate (TTM)	Sigma	Cat#323446
BrdU	Beyotime	Cat#ST1056
DAPI Staining Solution	Beyotime	Cat#C1005
Propidium Iodide solution (PI)	Wanleibio	Cat#WLA010a
Profinity^TM^ IMAC resin	Biorad	Cat#1560121
Imadazole	Sigma	Cat#I2399
Radio Immunoprecipitation Assay (RIPA) lysis buffer	Beyotime	Cat#P0013C
Trizol	Life Technology Co	Cat#15596026
absolute ethanol	Supelco	Cat#1.07017
RNase A	Roche	Cat#10109142001
collagenase	Life-iLab Biotech	Cat#AC15L141
BSA	Sigma	Cat#V900933
Proteinase inhibitor	Thermo Fisher Scientific	Cat#89900
Proteinase K	MERCK	Cat#39450-01-6
Triton X-100	Sigma	Cat#T9284
Dimethyl Sulphoxide (DMSO)	Biosharp	Cat#BS087
lipofectamine™ 2000	Thermo Fisher Scientific	Cat#11668-019
1-Phenyl-2-thioourea (PTU)	Sigma	Cat#2954-52-1

**Critical commercial assays**		

M-MLV Reverse-Transcript Kit	Promega	Cat#M1701
CellsDirect^TM^ One-Step qRT-PCR Kit	Thermo Fisher Scientific	Cat#11753-100
mMessage mMachine kit	Ambion	Cat#AM1344
Dual-Luciferase Reporter Assay System	Promega	Cat#E1910
Transcript T7 High Yield Transcription kit	Thermo Fisher Scientific	Cat#K0441
DIG RNA Labeling Mix	Roche	Cat#11277073910

**Experimental models: Cell lines**		

Human leukemia cell line K562	ATCC	Cat#CCL-243
human embryonic kidney (HEK) 293 cell	ATCC	Cat#CRL-3216
human Umbilical Vein Endothelial Cells (HUVECs)	ATCC	Cat#CRL-1730
human Umbilical Vein Endothelial Cells (HUVECs) ShFOXM1	This paper	N/A

**Experimental models: Organisms/strains**		

Zebrafish strain AB (WT)	This paper	N/A
Zebrafish: *Tg*(*runx1*:GFP)	Zhang et al.^78^	N/A
Zebrafish: *Tg*(*runx1*:GFP/*flk1*:mcherry)	Zhang et al.^78^	N/A
Zebrafish: *cox17*^-/-^	Sun et al.; Li et al.^25^^,^^49^	N/A
Zebrafish: *atp7b*^-/-^	Zhang et al.^11^	N/A
Zebrafish: *atp7a*^-/-^	Zhao et al.^14^	N/A
Zebrafish: *tuba1a*^-/-^	This paper	N/A
Zebrafish: *stmn4*^-/-^	This paper	N/A
Zebrafish: *foxm1*^-/-^	This paper	N/A

**Oligonucleotides**		

gRNA targeting sequence for XX, see Table S2	This paper	N/A
Morpholinos sequences for XX, see Table S4	Gene Tools	N/A
Primers for mutation target detection, see Table S3	This paper	N/A
shRNA targeting sequence for XX, see Table S5	This paper	N/A
Probe for WISH, see Table S6	This paper	N/A
Primers for qRT-PCR, see Table S7	This paper	N/A
Primers for mRNA synthesis, see Table S8	This paper	N/A
Primers for promoter activity assays, see Table S9	This paper	N/A
Primers for ChIP-qPCR, see Table S10	This paper	N/A

**Deposited data**		

RNA sequencing	This paper	https://doi.org/10.6084/m9.figshare.22177763.v2

**Software and algorithms**		

ImageJ	ImageJ	https://imagej.net/software/imagej/
GraphPad Prism 8.00	Graphpad	https://www.graphpad.com/
Statistic Package for Social Science (SPSS) 19.0	Spss	https://www.concordia.ca/it/services/spss.html

### Resource availability

#### Lead contact

Further information and requests for resources should be directed to and will be fulfilled by the lead contact, Jing-Xia Liu (ichliu@mail.hzau.edu.cn).

#### Materials availability

All materials generated in this study are available from the lead contact without restriction.

### Experimental model and subject details

#### Zebrafish husbandry and lines

All fish used in this study were reared and maintained in the Zebrafish Aquaculture Core Facility at College of Fisheries, Huazhong Agricultural University under 14 h light / 10 h dark cycle and 28 °C ± 0.5 °C and according to standard procedures, as we performed previously.^79^ Zebrafish is a gonochoristic species, and sex is determined by both internal and external factors.^80^ Natural eggs were obtained by mating a certain ratio of male and female zebrafish (male: female, 3: 2), and maintained at 28.5 °C in an incubator for development. The ages of the embryos and larvae were expressed by hpf or dpf. Fish used in this study were wild-type AB, TL lines and mutant lines. transgenic lines: *Tg*(*runx1*:GFP)^78^ and *Tg*(*runx1*:GFP/*flk1*:mcherry)^78^; and mutant line *cox17*^*−/−*^,^25^^,^^49^ *atp7b*^*−/−*^,^11^ *atp7a*^*−/−*^,^26^ as well as the mutants constructed in this study, including *tuba1a*^*−/−*^, *stmn4*^*−/−*^, and *foxm1*^*−/−*^. All zebrafish maintenance and experiments were conducted in accordance with the Guidelines for Experimental Animals approved by the Institutional Animal Care and Use Ethics Committee of Huazhong Agricultural University (permit number HZAUFI-2016-007).

#### Cell lines

Human leukemia cell line K562, human embryonic kidney (HEK) 293 cell and human Umbilical Vein Endothelial Cells (HUVECs) were purchased from American Type Culture Collection (Manassas, USA), and were cultured at 37 °C in a humid atmosphere of 95% air and 5% CO2 in an incubator, with HEK293T cells in Dulbecco’s modified Eagle’s medium (DMEM) (Gibco, USA) with 10% heat-inactivated fetal bovine serum (FBS, Gibco, USA), while K562 cells and HUVECs in RPMI (Roswell Park Memorial Institute) Medium (Gibco, USA) with 10% heat-inactivated FBS (Gibco, USA). As well as the shFOXM1 constructed in this study, and was cultured at 37 °C in a humid atmosphere of 95% air and 5% CO2 in an incubator, in RPMI (Roswell Park Memorial Institute) Medium (Gibco, USA) with 10% heat-inactivated FBS (Gibco, USA).

### Method details

The full names and their abbreviations for genes mentioned in this study are listed in Table S1.

#### Morpholinos and Cas9/gRNA

In this study, the CRISPR/Cas9 genome edition system was used to construct *tubulin alpha 1a* (*tuba1a*), *stathmin-like 4* (*stmn4*), and *forkhead box M1* (*foxm1*) zebrafish mutants. The guide RNAs (gRNAs) were designed to target the first exon of the aforementioned genes by ZiFiT Targeter Version 4.2 (http://zifit.partners.org/ZiFiT/CSquare9Nuclease.aspx). Sequences of gRNAs are listed in Table S2. The genotyping assays of *tuba1a*, *stmn4* and *foxm1* heterozygote and homozygous mutants were performed in the F1 and F2 generation from the fundamental fish with genome edition at targeted loci in germline as reported previously,^25^^,^^81^ and the genotyping primers are listed in Table S3. The phenotypes of mutants at different developmental stages were observed and photographed. *Tuba1a* knockout homozygous mutants were embryonic lethal from 6 dpf (days post fertilization), all died aroud 8-9 dpf, and *tuba1a*^*−/−*^ mutants were one-by-one genotyped for dead fish and in whole-mount *in situ* hybridization (WISH) assays in this study. The morpholinos (MOs), including *tuba1a*-MO, *stmn4*-MO, *tubb5* (*tubulin, beta 5*)-MO, *tmsb2* (*thymosin beta 2*)-MO, *foxm1*-MO, and *p53*-MO were purchased from Gene Tools, LLC (Philomath, Oregon, USA) and their sequences are listed in Table S4. In all experiments, the MOs were injected into one-cell stage embryos, with the MO doses of *tuba1a*, *stmn4*, *tubb5*, *tmsb2* and *foxm1* at 0.9 mM, 0.9 mM, 0.9 mM, 0.9 mM and 0.6 mM, respectively, and MO dose of *p53* at 1.0 mM.

Studies shown that the off-target effects of MOs are mediated by P53 activation,^35^^,^^36^^,^^37^ and co-injection of *p53* MO is a well-known standard to estimate whether the tested morphology or molecular defects are derived from the ameliorate apoptosis induced by MO off-targets, from P53 activation, or from the down-regulation of gene expression.

#### ShFOXM1 cell lines construction

*FOXM1* shRNA (Table S5) was synthesized to target the different loci of gene *FOXM1*. Lentiviruses for different shFOXM1 or shGFP controls were generated by transfecting HEK293T cells with a transducing vector as well as packaging vectors pMD.2 and pspAX.^81^ After transfection for 48 h, viral particles in the medium were harvested and transduced into target cells (HUVECs). The virus infected cells were selected with puromycin (1 mg/mL) and verified by cell-directed qRT-PCR to check the FOXM1 expression. The cells infected with specific shFOXM1 lentiviruses and exhibiting significantly reduced expression of FOXM1 were expanded and maintained as stable shFOXM1-knockdown cell lines.^12^

#### Drug exposure and phenotype observation

The Cu ion (Cu) exposure solution was prepared as we performed recently.^82^ Briefly, embryos were exposed to Cu^2+^ (CuSO_4_·5H_2_O) (Sigma, Cat#61245) before sphere stage at 3.9 μM Cu concentration.^38^ Elesclomol (Selleck, Cat#S1052) and was dissolved in Dimethyl Sulphoxide (DMSO) (Biosharp, Cat#BS087) at 10 mM for stock solutions, and tetrathiomolybdate (TTM) (Sigma, Cat#323446) were dissolved in DMSO at 20 mM for stock solutions. 100 nM elesclomol was added to the Cu treated groups,^29^^,^^83^ and 200 μM TTM added to the control groups.^29^ The embryos were collected at the indicated stages. Embryos from the control and the treated groups were observed and photographed using a light microscope (Leica M205FA) to examine their embryonic morphology. The embryos were collected at the indicated stages for experiments as indicated in Schema 1.

#### Cellular Cu ion level assays

*Tg*(*runx1*:GFP) embryos before sphere stage were stressed with 3.9 μM Cu to 33 hpf. After treatment, the embryos were homogenized, followed by cell filtration. Cells of embryos were collected with ice-cold PBS. Next, 500 μL ice-cold PBS and 0.5 μL Cu ion probe (Rhodamine type Cu ion probe, labelling intracellular Cu ion, kindly provided by Pro. Gao, Hebei University, Baoding 071002, P. R. China) was added to each sample, followed by incubation in the dark for 2 min at 28 °C. Analysis of the cellular Cu ion in Cu stressed *runx1*GFP^+^ cells using FACS (CytoFLEX S, Beckman Coulter, USA).

For the whole mount Cu probe staining, the Cu-treated and the control Tg(*runx1*:GFP) embryos at 33 hpf were separately incubated with 500 μL ice-cold PBS and 0.5 μL Cu ion probe for 15 min at 28 °C. After washing with PBS for 2-3 times, the staining embryos were observed with a Leica TCS SP8 confocal laser microscope (Wetzlar, Germany).

#### Inductively coupled plasma mass spectrometry (ICP-MS)

Cu content in the embryos was determined by inductively coupled plasma–mass spectrometry (ICP-MS). Embryos were exposed to TTM, or Cu, or Cu + elesclomol beginning at the fertilization stage, the embryos were collected at 33 hpf, and each group was exposed in three independent replicates. Samples were digested in nitric acid (HNO3, 70%, MOS grade) for 2 h. Subsequently, each sample was heated (150 °C) for evaporation and made up to 5 mL final volume by addition of 2% nitric acid for analysis.

#### Cu ion affinity binding

Profinity^TM^ IMAC resin (Biorad, Cat#1560121) was washed and loaded with metals (0.2 M of either Fe^3+^ (Ammonium iron (III) citrate, Ammonium ferric citrate)) or Cu^2+^ (CuSO_4_) based on the manufacturers protocol. Human leukemia K562 cells with or without Cu stress were lysed with Radio Immunoprecipitation Assay (RIPA) buffer (Beyotime, Cat#P0013C), and the total lysates loaded on the column. Column was washed three times with 1xPBS and eluted with 300 mM Imadazole (elution buffer) (Sigma, Cat#I2399). Eluted proteins were analyzed by western blot (WB) analysis.

#### RNA-sequencing (RNA-Seq) and analysis

The Tg(*runx1*:GFP) embryos were collected and homogenized, followed by sorting the cells, control *runx1*GFP^+^ cells and Cu-treated *runx1*GFP^+^ cells, respectively, at 33 hpf, into the lysate by fluorescence activating cell sorting (FACS) (BD FacsAria SORP, 650110M3, BioDot, American), and the lysate for RNA extraction and RNA-sequencing (RNA-Seq). RNA-Seq was performed in the Novogene (Beijing, China) using an Illumina HiSeqTM as reported in our previous study.^82^ The zebrafish reference genome GRCz11 were used for mapping and algorithm to determine differentially expressed genes (DEGs), and genes with significant alterations due to Cu stress (adjusted *P* < 0.05) were defined as DEGs and subjected to functional annotation analysis. Enriched KEGG (Kyoto Encyclopedia of Genes and Genomes) pathway annotation analysis was conducted for each of the treatments using KOBAS v 2.0, and a cut-off of *P* value < 0.05. GO (Gene Ontology) analysis was conducted by GOseq Release 2.12 (*P* value < 0.05). Hierarchical clustering was performed by TIGR Multi experiment Viewer (MeV) to generate different heatmaps as we reported previously.^82^ The defined DEGs and the analyzed GO and KEGG pathway data were available at Figshare, and the DOIs are listed in the key resources table.

#### Whole-mount *in situ* hybridization (WISH)

Probes for zebrafish *tuba1a*, *stmn4*, *tubb5*, *foxm1*, and *ctr1* were amplified from cDNA pools using primers shown in Table S6. Probes for zebrafish *runx1*, *cmyb*, *pax2a* (+*myoD*) *scl*, *lmo2*, *gata1a* and *mpx* have been reported previously.^49^^,^^84^ The anti-sense RNA probes labeled with digoxygenin (Roche, Cat#11277073910) were used to verify the expression of target genes in the whole-mount embryos with 2–3 biological replicates. WISH embryos in each group were photographed with a Leica M205FA stereomicroscope and a representative image in each group was shown. *tuba1a*^-/-^ mutant was homozygously lethal from 6 dpf, and the WISH embryos were identified by one-by-one genotyping.

#### Quantitative RT-PCR analysis

Zebrafish embryos and mammalian cells were collected separately at indicated stages, and total RNA was isolated from ∼50 whole embryos/sample or cell samples using Trizol reagent (Life Technology Co, Cat#15596026). cDNA was synthesized using a M-MLV Reverse-Transcript Kit (Promega, Cat#M1701), and the qRT-PCR amplification was performed for 40 cycles at 95 °C for 10 s, 59 °C for 10 s, and 72 °C for 10 s. Each sample was biologically repeated in triplicate. Differences were calculated by the ΔΔCt comparative quantization method using *18s* as an internal control.^38^^,^^81^^,^^85^ In this study, qRT-PCR analysis was performed for human genes of *BAX*, *SHC1*, *CDC6*, *CKS1B*, *CDC25A*, *MYCA*, *CYCLINA1*, *CYCLINA2*, *CYCLINB*, *CYCLIND1*, *CYCLINE1*, *CYCLING2*, *CDC25B*, *CDK1*, *CENPF*, *P130*, *FOXM1*, *TMSB2*, *TUBA1A*, *STMN4*, and *TUBB5* using the primers listed in Table S7.

#### One step cell-direct qRT-PCR

One step cell-direct qRT-PCR was performed as reported recently.^34^^,^^81^ Briefly, the Tg(*runx1*:GFP) embryos were collected and homogenized, followed by sorting the different cell groups, *runx1*GFP^+^ and *runx1*GFP^-^ cells, with or without Cu stress, into the lysate of the CellsDirect^TM^ One-Step qRT-PCR Kit (Thermo Fisher Scientific, Cat#11753-100) by FACS (BD FacsAria SORP, 650110M3, BioDot, American). Primer sequences for the tested genes are shown in Table S7, including zebrafish genes (*olig2*, *fabp2*, *mbp*, *myod*, *runx1*, *cmyb*, *gata2b*, *ctr1*, *cyclina1*, *cyclina2*, *cyclinb*, *cyclind1*, *cyclinge1*, *cycling2*, *cenpf*, *atm*, *p130*, *cdc25b*, *cdk1*, *myh7l*, *myh7*, *map1aa*, *stmn4*, *tuba1a*, *tubb5*, *tmsb2*, *foxm1*, *hsf1*, *sp1*). The qPCR as described above, and each sample was biologically repeated at least three times. Differences were calculated by the ΔΔCt comparative quantization method using *18s* as an internal control.

#### mRNA synthesis and injection

For mRNA preparation, capped mRNAs were synthesized using the mMessage mMachine kit (Ambion, Cat#AM1344) as instructed by the manufacturer. The synthesized mRNAs were diluted into different concentrations and injected into one-cell stage embryos as reported previously,^85^ and the mRNA concentrations of *foxm1*, *tuba1a*, and *stmn4 both* at 200 ng/μL. Full-length zebrafish *foxm1*, *tuba1a*, and *stmn4* were amplified from cDNA pools by using the appropriate set of primers shown in Table S8.

#### Fluorescence microscopy observation

Embryos were anesthetized with a low dose of tricaine and mounted on dishes with 1% low-melting agarose for observing the *flk1*^*+*^*runx1*^*+*^ double positive cells in different treated *Tg*(*runx1*:GFP/*flk1*:mcherry) embryos. Confocal images were acquired with a Leica TCS SP8 confocal laser microscope (Wetzlar, Germany). The number of HSPCs was counted based on yellow particles obtained by the overlap of the red and green fluorescence in the cells, and the fluorescence intensity in the positive cells was analyzed by Image J software.

#### Cell cycle analysis

The Cu stressed Tg(*runx1*:GFP) embryos and the control embryos were homogenized separately and filtered, followed by sorting the *runx1*GFP^+^ cells by FACS (BD FacsAria SORP 650110M3 BioDot, American), and the collected cells were fixed in the pre-cooled 75% absolute ethanol for over 2 h. Then, after washing with PBS, 100 μL of RNase A was added to the cells for 30 min at 28 °C. Next, the DNA was stained with 500 μL DNA Prep stain (Propidium iodide solution, PI) (Wanleibio, Cat#WLA010a) and incubated in the dark for 30 min at 28 °C. Finally, the cellular DNA content was analyzed using the CytoFLEX Flow Cytometer (Beckman Coulter, USA), followed by estimating the percentage of cells in the G1 phase, S phase, and G2/M phase.

Additionally, Tg(*runx1*:GFP) embryos at 33 hpf and 72 hpf were fixed with 4% PFA overnight at 4 °C, followed by dehydration with methanol, incubation at -20 °C overnight, and then treatment with 1 mg/mL collagenase (Life-iLab Biotech, Cat#AC15L141) for 45 min at RT. After washing with PBST, the embryos were blocked in 4% BSA for 1 h, followed by incubation separately with the primary antibodies of α-Tubulin (1:300) and rabbit anti GFP-Tag pAb (1:200) at 4 °C for 72 h and 48 h respectively, then with the secondary antibodies of Alexa Fluor 555-conjugated Goat Anti-mouse IgG (H+L) (1:200) and Goat Anti-Rabbit IgG FITC (H+L) (1:200) at RT for 2 h. The cell nuclei were stained with 40, 6-diamidino-2-phenylindole (DAPI, 5 μg/mL). Finally, confocal images were acquired with a Leica TCS SP8 confocal laser microscope (Wetzlar, Germany).

#### Plasmids Construction

Briefly, genomic DNA was extracted from ∼ 50-60 embryos or HEK293T cells using the ammonium acetate method and quantified using a Nanodrop spectrophotometer (Thermo Fisher) for amplification of gene promoter fragments. The primers in Table S9 were used for amplification of 5′ unidirectional human *TUBA1A* promoter, including −1950; human *STMN4* promoter, including −1959; human *FOXM1* promoter, including −2176, −1086, −755, −317 and −106; zebrafish *tuba1a* promoter, including −1950; zebrafish *stmn4* promoter, including −2024; zebrafish *foxm1* promoter, including −2113, followed by cloning them separately into the pGL3 vector. Full-length human *ATOX1*, *FOXM1*, *SP1*, *HSF1* and zebrafish *atox1*, *foxm1*, *sp1*, *hsf1* were amplified from cDNA pools using the appropriate sets of primers (Table S9). Human *ATOX1*, *FOXM1*, *SP1*, *HSF1* and zebrafish *atox1*, *foxm1*, *sp1*, *hsf1* were sub-cloned into the pCGN-HAM and pCMV-Myc vectors, respectively. All plasmids were verified by sequencing.

#### Western blot (WB)

Embryos and cells were homogenized using RIPA lysis buffer with proteinase inhibitor (Thermo Fisher Scientific, Cat#89900), followed by adding an appropriate SDS-PAGE loading buffer, boiling, and loading for polyacrylamide gel electrophoresis. After separation of an almost equal amount of protein in each line, the separated protein was transferred to polyvinylidene fluoride microporous membrane (PVDF) (Bio-Rad Laboratories, Hercules, CA, USA). Next, the blots were blocked with 5% skim milk in TBS containing 0.1% Triton X-100, followed by incubation first with the primary antibodies against TUBA1A, STMN4, FOXM1, CDK1, CCNB1, CDC25B, SP1, Myc-Tag, HA-Tag, GAPDH or β*-*ACTIN, respectively, in a 1:1000 dilution, and then with secondary antibodies (1:5000), the blots were visualized using enhanced chemiluminescence (Bio-Rad Laboratories, Hercules, CA, USA) and photographed by the Amersham Imager 600 analyzer. ImageJ software (NIH, Bethesda, Maryland) was used for quantifying the protein levels based on the band density from Western blot analysis.

#### BrdU labeling and immunofluorescence

The Cu-treated and the control Tg(*runx1*:GFP) embryos at 31 hpf and 70 hpf, respectively, were injected with BrdU (10 mM; Beyotime, Cat#ST1056) peritoneally, followed by incubation for 2 h and fixing in 4% paraformaldehyde (PFA). Next, the fixed embryos were dehydrated with methanol and kept at -20 °C overnight, followed by rehydration and treating with 1 mg/mL collagenase for 45 min at RT. After washing with PBST, the embryos were incubated with 2.4 mol/L HCl for 1 h at RT, rinsed with PBST and then blocked in 4% BSA for 1 h. Finally, the embryos were stained with BrdU Mouse mAb (1:200) and Rabbit anti GFP-Tag pAb (1:200) antibodies according to the manufacturer’s protocol. Alexa Fluor 555-conjugated Goat Anti-mouse IgG (H+L) (1:200) and Goat Anti-Rabbit IgG FITC (H+L) (1:200) secondary antibodies were used for double staining. For β-Tubulin and PH3 immunofluorescence, the FACS cells and the whole embryos were stained with β-Tubulin or PH3 antibodies in a 1:200 dilution, respectively, followed by the secondary antibodies with Alexa Fluor 488 in a 1:500 dilution.

Additionally, for HSF1 or SP1, with Cu immunofluorescence together, the K562 cells were stained with HSF1 or SP1 antibodies in a 1:200 dilution, respectively, followed by the secondary antibodies with Alexa Fluor 488 in a 1:500 dilution. Next, 500 μL ice-cold PBS and 0.5 μL Cu ion probe was added to each sample, followed by incubation in the dark for 2 min at 28 °C. The nuclei were stained with DAPI. The images of immunofluorescence were obtained with a Leica TCS SP8 confocal laser microscope (Wetzlar, Germany).

#### ChIP-qPCR

HEK293T cells were cultured as described above. Formaldehyde (Fisher) was added to the culture medium to a final concentration of 1%, and after incubation for 8 min at RT, cross-linking was terminated by adding glycine at a final concentration of 0.125 M, then, followed by further incubation for 8 min. After two washes with 1xPBS, the cells were centrifuged to obtain the pellet, followed by successive treatment for 10 min in lysis buffer 1 (50 mM HEPES-KOH pH 7.5, 140 mM NaCl, 1 mM EDTA, 10% glycerol, 0.5% NP-40, 0.25% Triton X-100) and lysis buffer 2 (10 mM Tris-HCl pH 8.0, 200 mM NaCl, 1 mM EDTA, 0.5 mM EGTA). Next, the pellet was suspended in 1 mL nucleus lysis buffer 3 (10 mM Tris-HCl pH 8, 100 mM NaCl, 1 mM EDTA, 0.5 mM EGTA, 0.1% Na-Deoxycholate, 0.5% N-lauroylsarcosine), followed by sonication to obtain ∼ 200-500 bp chromatin DNA fragments.

After sonication, the input control, SP1, MYC-SP1 (SP1), MYC-HSF1 (HSF1), and IgG (negative control) ChIP groups were centrifuged for 10 min at 18,000 g and 1% of Triton X-100 was added to the supernatant. Prior to the immunoprecipitation, 50 μL of protein A+G agaroses (Beyotime, Cat#P2055) for each reaction were washed twice with 0.5% BSA, and the the agaroses were resuspended in 250 μL of 0.5% BSA with 5 μg of each antibody. Agaroses with each antibody were separately rotated for at least 6 h at 4 °C and then washed twice with 0.5% BSA, next, cell lysates were added, and then the mixtures were incubated at 4 °C overnight. Beads were washed with buffer 1 (20 mM Tris-HCl pH8, 150 mM NaCl, 2 mM EDTA, 0.1% SDS, 1% Triton X-100) one or two times, then buffer 2 (20 mM Tris-HCl pH8, 500 mM NaCl, 2 mM EDTA, 0.1% SDS, 1% Triton X-100) one or two times, then with buffer three (10 mM Tris-HCl pH8, 250 nM LiCl, 2 mM EDTA, 1% NP40) one or two times, and finally resuspended in 200 μL elution buffer (50 mM Tris-HCl, pH 8.0, 10 mM EDTA and 0.5-1% SDS) by heating at 65 °C for 30 min in a shaking heat block for at least 6 h^10^^,^^81^ 50 μL of cell lysates prior to addition to the beads was kept as input. After the ChIP DNA was recovered by phenol/chloroform/isoamylalcohol (25:24:1) extraction and precipitated by ethanol, the pellet was re-suspended in sterile water and used as a template for qPCR. The tested genes and their primers used for ChIP-qPCR are listed in Table S10, and qPCR and data analysis as described above.

#### Luciferase reporter assay

In this study, the reporter vectors of *FOXM1*, *STMN4*, *TUBA1A*, and different truncated mutants of *FOXM1* were used for luciferase reporter assays as described previously.^10^^,^^11^^,^^81^ All plasmids were transiently transfected into HEK293T cells using lipofectamine™ 2000 (Thermo Fisher Scientific, Cat#11668-019) following the manufacture’s protocol. After harvesting the transfected cells, the luciferase activity assays were performed using the Dual-Luciferase Reporter Assay System (Promega, Cat#E1910), and the luciferase assay data were analyzed using GraphPad Prism 8.0.

### Quantification and statistical analysis

#### Statistical analysis

A sample size larger than 10 embryos (n > 10) was used for different experiments in each group, with 2-3 biological replicates for each test. Percentage analysis of the results among different groups was performed using hypergeometric distribution in the R-console software.^86^ For WISH results in different experimental groups, the number in the figure was shown as N_changed_/N_total_, where N_changed_ indicates the number of embryos exhibiting reduced or increased expression, and N_total_ indicates the total number of embryos in a group; the number in the figure for the control groups was shown as N_normal_/N_total_, where N_normal_ indicates the number of embryos exhibiting normal expression and N_total_ indicates the total number of embryos in a group. The signal area of representative images was calculated in each representative embryo (N ≥ 3) in WISH by Image J software (NIH, Bethesda, Maryland) (Firstly, the WISH’s picture was converted to type 8-bit, then the area was adjusted by Image Adjust Brightness/Contrast, then Set Scale, and finally the measure) and shown as scatter plots, with each dot indicating the signal level of a representative image in an individual embryo in each group, and the data were analyzed using *t-test* by GraphPad Prism 8.00 software as we performed recently.^10^^,^^11^ Additionally, the percentage of the WISH results was determined by hypergeometric distribution analysis using the R-console software.

The number of HSPCs and HSPC proliferation were quantified from the confocal images of the AGM (470 μm×470 μm) and CHT (470 μm×470 μm) with z-stacks spanning the entire trunk thickness, and the number of *flk1*^*+*^*runx1*^*+*^ and anti-GFP^+^ (*runx1*^+^) BrdU^+^ cells were manually counted using ImageJ software (NIH). At least ten randomly selected units were analyzed for each control group and experimental group. The number of PH3^+^ HSPCs was quantified in a similar way as described above. Also, the fluorescence intensity of cytoskeleton in a single cell was also quantified from the confocal images of the positive β-Tubulin signals in *runx1*GFP^+^ cell (22.15 μm×22.15 μm) with z-stacks spanning the entire cell thickness, and the fluorescence intensity of cytoskeleton was measured using ImageJ. The statistical data of the signal area and fluorescence level in different samples were analyzed using *t-test* by GraphPad Prism 8 software, with each dot representing the signal level in an individual embryo in each group.

Statistical data of cell cycle were processed by GraphPad Prism 8 software. Each dot represents the cell percentage in each cell cycle phase. The qRT-PCR data were analyzed by one-way analysis of variance (ANOVA) and *post hoc* Tukey’s test in the Statistic Package for Social Science (SPSS) 19.0 software, with each dot representing one repeat. The statistical analysis for luciferase reporter assay results was performed using GraphPad Prism 8 software (unpaired t-test) (GraphPad Software Inc). Data were presented as mean ± SD, ∗*P* < 0.05, ∗∗*P* < 0.01, ∗∗∗*P* < 0.001.

## Data Availability

• The RNA sequencing data have been deposited at Figshare and are publicly available as of the date of publication. DOIs are listed in the key resources table. • This paper does not report original code. • Any additional information required to reanalyze the data reported in this paper is available from the lead contact upon request.

## Supplemental information

Document S1. Figures S1–S11 and Schema 1

Table S1. Full names for genes tested in this study, related to all Figure

Table S2. sequences of gRNA, related to Figures 4 and 5

Table S3. sequences of primers for mutation target detection, related to Figure 4 and Figure 5

Table S4. sequences of Morpholinos, related to Figure 4 and Figure 5

Table S5. sequences of primers used for shRNA, related to Figure 7

Table S6. sequences of primers used for amplifying probes for WISH, related to all Figure

Table S7. sequences of primers used for qRT-PCR, related to Figure 1 and Figure 7 and Figure 8

Table S8. sequences of primers for mRNA synthesis, related to Figure 4-6

Table S9. sequences of primers used for promoter activity assays, related to Figure 6 and Figure 8

Table S10. sequences of primers used for ChIP-qPCR, related to Figure 8

Table S11. GO enrichment of down-regulated DEGs in Cu2+ stressed runx1 GFP positive cells, related to Figure 4

Table S12. cytoskeleton, myeloid and erythrocyte genes expressed in Cu2+ stressed runx1 GFP positive cells, related to Figure 4

Table S13. GO enrichment of up-regulated DEGs in Cu2+ stressed runx1 GFP positive cells, related to Figure 4

Table S14. The most enriched KEGG terms in runx1 GFP positive cells from control and Cu2+ stressed embryos at 33 hpf, related to Figure 4

Table S15. KEGG enrichment of down-regulated DEGs in Cu2+ stressed runx1 GFP positive cells at 33 hpf, related to Figure 4

Table S16. KEGG enrichment of up-regulated DEGs in Cu2+ stressed runx1 GFP positive cells at 33 hpf, related to Figure 4

Table S17. Differentially expressed genes (DEGs) in Cu2+ stressed runx1 GFP positive cells at 33 hpf, related to Figure 4

Table S18. Down-regulated genes (DEGs) in Cu2+ stressed runx1 GFP positive cells at 33 hpf, related to Figure 4

Table S19. Up-regulated genes (DEGs) in Cu2+ stressed embryos at 24 hpf, related to Figure 5

Table S20. Down-regulated genes (DEGs) in Cu2+ stressed embryos at 24 hpf, related to Figure 5

Table S21. vessel genes expressed in Cu2+ stressed embryos, related to Figure 5

## References

[1] Shimizu M. (1979). Clinical results on the use of human ceruloplasmin in aplastic anemia. Transfusion.

[2] Bustos R.I., Jensen E.L., Ruiz L.M., Rivera S., Ruiz S., Simon F., Riedel C., Ferrick D., Elorza A.A. (2013). Copper deficiency alters cell bioenergetics and induces mitochondrial fusion through up-regulation of MFN2 and OPA1 in erythropoietic cells. Biochem. Biophys. Res. Commun..

[3] Halfdanarson T.R., Kumar N., Li C.Y., Phyliky R.L., Hogan W.J. (2008). Hematological manifestations of copper deficiency: a retrospective review. Eur. J. Haematol..

[4] Kim S., Freeland-Graves J.H., Babaei M., Sachdev P.K., Beretvas S.N. (2019). Quantifying the association between acute leukemia and serum zinc, copper, and selenium: a meta-analysis. Leuk. Lymphoma.

[5] Demir C., Demir H., Esen R., Sehitogullari A., Atmaca M., Alay M. (2011). Altered serum levels of elements in acute leukemia cases in Turkey. Asian Pac. J. Cancer Prev. APJCP.

[6] Zuo X.L., Chen J.M., Zhou X., Li X.Z., Mei G.Y. (2006). Levels of selenium, zinc, copper, and antioxidant enzyme activity in patients with leukemia. Biol. Trace Elem. Res..

[7] Orkin S.H., Zon L.I. (2008). Hematopoiesis: an evolving paradigm for stem cell biology. Cell.

[8] Goessling W., North T.E., Loewer S., Lord A.M., Lee S., Stoick-Cooper C.L., Weidinger G., Puder M., Daley G.Q., Moon R.T., Zon L.I. (2009). Genetic interaction of PGE2 and Wnt signaling regulates developmental specification of stem cells and regeneration. Cell.

[9] Li P., Lahvic J.L., Binder V., Pugach E.K., Riley E.B., Tamplin O.J., Panigrahy D., Bowman T.V., Barrett F.G., Heffner G.C. (2015). Epoxyeicosatrienoic acids enhance embryonic haematopoiesis and adult marrow engraftment. Nature.

[10] Jin X., Liu W., Miao J., Tai Z., Li L., Guan P., Liu J.X. (2021). Copper ions impair zebrafish skeletal myofibrillogenesis via epigenetic regulation. Faseb. J..

[11] Zhang T., Guan P., Liu W., Zhao G., Fang Y., Fu H., Gui J.F., Li G., Liu J.X. (2020). Copper stress induces zebrafish central neural system myelin defects via WNT/NOTCH-hoxb5b signaling and pou3f1/fam168a/fam168b DNA methylation. Biochim. Biophys. Acta. Gene Regul. Mech..

[12] Tai Z., Li L., Zhao G., Liu J.X. (2022). Copper stress impairs angiogenesis and lymphangiogenesis during zebrafish embryogenesis by down-regulating pERK1/2-foxm1-MMP2/9 axis and epigenetically regulating ccbe1 expression. Angiogenesis.

[13] Kondera E., Witeska M. (2013). Cadmium and copper reduce hematopoietic potential in common carp (Cyprinus carpio L.) head kidney. Fish Physiol. Biochem..

[14] Zhao G., Sun H., Zhang T., Liu J.X. (2020). Copper induce zebrafish retinal developmental defects via triggering stresses and apoptosis. Cell Commun. Signal..

[15] Hatori Y., Lutsenko S. (2013). An expanding range of functions for the copper chaperone/antioxidant protein Atox1. Antioxidants Redox Signal..

[16] Matson Dzebo M., Blockhuys S., Valenzuela S., Celauro E., Esbjörner E.K., Wittung-Stafshede P. (2018). Copper chaperone Atox1 interacts with cell cycle proteins. Comput. Struct. Biotechnol. J..

[17] Davies K.M., Mercer J.F.B., Chen N., Double K.L. (2016). Copper dyshomoeostasis in Parkinson's disease: implications for pathogenesis and indications for novel therapeutics. Clin. Sci..

[18] de Bie P., Muller P., Wijmenga C., Klomp L.W.J. (2007). Molecular pathogenesis of Wilson and Menkes disease: correlation of mutations with molecular defects and disease phenotypes. J. Med. Genet..

[19] Horn N., Wittung-Stafshede P. (2021). ATP7A-Regulated enzyme Metalation and trafficking in the Menkes disease puzzle. Biomedicines.

[20] Bandmann O., Weiss K.H., Kaler S.G. (2015). Wilson's disease and other neurological copper disorders. Lancet Neurol..

[21] Stremmel W., Merle U., Weiskirchen R. (2019). Clinical features of Wilson disease. Ann. Transl. Med..

[22] Chen J., Jiang Y., Shi H., Peng Y., Fan X., Li C. (2020). The molecular mechanisms of copper metabolism and its roles in human diseases. Pflügers Archiv.

[23] Myint Z.W., Oo T.H., Thein K.Z., Tun A.M., Saeed H. (2018). Copper deficiency anemia: review article. Ann. Hematol..

[24] Singh R.P., Jeyaraju D.V., Voisin V., Hurren R., Xu C., Hawley J.R., Barghout S.H., Khan D.H., Gronda M., Wang X. (2020). Disrupting mitochondrial copper distribution inhibits leukemic stem cell self-renewal. Cell Stem Cell.

[25] Sun H., Chen M., Wang Z., Zhao G., Liu J.X. (2020). Transcriptional profiles and copper stress responses in zebrafish cox17 mutants. Environ. Pollut..

[26] Zhao G., Zhang T., Sun H., Liu J.X. (2020). Copper nanoparticles induce zebrafish intestinal defects via endoplasmic reticulum and oxidative stress. Metallomics.

[27] Wattrus S.J., Smith M.L., Rodrigues C.P., Hagedorn E.J., Kim J.W., Budnik B., Zon L.I. (2022). Quality assurance of hematopoietic stem cells by macrophages determines stem cell clonality. Science.

[28] Lee J., Petris M.J., Thiele D.J. (2002). Characterization of mouse embryonic cells deficient in the ctr1 high affinity copper transporter. Identification of a Ctr1-independent copper transport system. J. Biol. Chem..

[29] Tsvetkov P., Coy S., Petrova B., Dreishpoon M., Verma A., Abdusamad M., Rossen J., Joesch-Cohen L., Humeidi R., Spangler R.D. (2022). Copper induces cell death by targeting lipoylated TCA cycle proteins. Science.

[30] Denoyer D., Masaldan S., La Fontaine S., Cater M.A. (2015). Targeting copper in cancer therapy: ‘Copper that Cancer’. Metallomics.

[31] Vermeulen K., Berneman Z.N., Van Bockstaele D.R. (2003). Cell cycle and apoptosis. Cell Prolif..

[32] Alenzi F.Q.B. (2004). Links between apoptosis, proliferation and the cell cycle. Br. J. Biomed. Sci..

[33] Zhou X.Y., Zhang T., Ren L., Wu J.J., Wang W., Liu J.X. (2016). Copper elevated embryonic hemoglobin through reactive oxygen species during zebrafish erythrogenesis. Aquat. Toxicol..

[34] Chen M., Luo Y., Xu J., Chang M.X., Liu J.X. (2019). Copper regulates the susceptibility of zebrafish larvae to inflammatory stimuli by controlling neutrophil/Macrophage survival. Front. Immunol..

[35] Ekker S.C., Larson J.D. (2001). Morphant technology in model developmental systems. Genesis.

[36] Plaster N., Sonntag C., Busse C.E., Hammerschmidt M. (2006). p53 deficiency rescues apoptosis and differentiation of multiple cell types in zebrafish flathead mutants deficient for zygotic DNA polymerase delta1. Cell Death Differ..

[37] Robu M.E., Larson J.D., Nasevicius A., Beiraghi S., Brenner C., Farber S.A., Ekker S.C. (2005). p53 activation by knockdown technologies. PLoS Genet..

[38] Zhang T., Xu L., Wu J.J., Wang W.M., Mei J., Ma X.F., Liu J.X. (2015). Transcriptional responses and mechanisms of copper-induced dysfunctional locomotor behavior in zebrafish embryos. Toxicol. Sci..

[39] Kwok C.T.D., Leung M.H., Qin J., Qin Y., Wang J., Lee Y.L., Yao K.M. (2016). The Forkhead box transcription factor FOXM1 is required for the maintenance of cell proliferation and protection against oxidative stress in human embryonic stem cells. Stem Cell Res..

[40] Hou Y., Li W., Sheng Y., Li L., Huang Y., Zhang Z., Zhu T., Peace D., Quigley J.G., Wu W. (2015). The transcription factor Foxm1 is essential for the quiescence and maintenance of hematopoietic stem cells. Nat. Immunol..

[41] Lozzio C.B., Lozzio B.B. (1973). Cytotoxicity of a factor isolated from human spleen. J. Natl. Cancer Inst..

[42] Lin M.I., Price E.N., Boatman S., Hagedorn E.J., Trompouki E., Satishchandran S., Carspecken C.W., Uong A., Dibiase A., Yang S. (2015). Angiopoietin-like proteins stimulate HSPC development through interaction with notch receptor signaling. Elife.

[43] Liao G.B., Li X.Z., Zeng S., Liu C., Yang S.M., Yang L., Hu C.J., Bai J.Y. (2018). Regulation of the master regulator FOXM1 in cancer. Cell Commun. Signal..

[44] Lou Q., Hu Y., Ma Y., Dong Z. (2016). Heat shock factor 1 induces crystallin-alphaB to protect against cisplatin nephrotoxicity. Am. J. Physiol. Ren. Physiol..

[45] Wang X., Zhang D., Cao M., Ba J., Wu B., Liu T., Nie C. (2018). A study on the biological function of heat shock factor 1 proteins in breast cancer. Oncol. Lett..

[46] Liu K., Ma R. (2021). MicroRNA-615-5p regulates the proliferation and apoptosis of breast cancer cells by targeting HSF1. Exp. Ther. Med..

[47] Lu S., Archer M.C. (2010). Sp1 coordinately regulates de novo lipogenesis and proliferation in cancer cells. Int. J. Cancer.

[48] Cigliano A., Pilo M.G., Li L., Latte G., Szydlowska M., Simile M.M., Paliogiannis P., Che L., Pes G.M., Palmieri G. (2017). Deregulated c-Myc requires a functional HSF1 for experimental and human hepatocarcinogenesis. Oncotarget.

[49] Li L., Chen M., Liu W., Tai P., Liu X., Liu J.X. (2022). Zebrafish cox17 modulates primitive erythropoiesis via regulation of mitochondrial metabolism to facilitate hypoxia tolerance. Faseb. J..

[50] Ge E.J., Bush A.I., Casini A., Cobine P.A., Cross J.R., Denicola G.M., Dou Q.P., Franz K.J., Gohil V.M., Gupta S. (2022). Connecting copper and cancer: from transition metal signalling to metalloplasia. Nat. Rev. Cancer.

[51] Lu Y., Pan Q., Gao W., Pu Y., Luo K., He B., Gu Z. (2022). Leveraging disulfiram to treat cancer: mechanisms of action, delivery strategies, and treatment regimens. Biomaterials.

[52] Heng Y.W., Koh C.G. (2010). Actin cytoskeleton dynamics and the cell division cycle. Int. J. Biochem. Cell Biol..

[53] Nunes V., Ferreira J.G. (2021). From the cytoskeleton to the nucleus: an integrated view on early spindle assembly. Semin. Cell Dev. Biol..

[54] Blajeski A.L., Phan V.A., Kottke T.J., Kaufmann S.H. (2002). G1 and G2 cell-cycle arrest following microtubule depolymerization in human breast cancer cells. J. Clin. Invest..

[55] Masaldan S., Clatworthy S.a.S., Gamell C., Smith Z.M., Francis P.S., Denoyer D., Meggyesy P.M., Fontaine S.L., Cater M.A. (2018). Copper accumulation in senescent cells: interplay between copper transporters and impaired autophagy. Redox Biol..

[56] Laoukili J., Kooistra M.R.H., Brás A., Kauw J., Kerkhoven R.M., Morrison A., Clevers H., Medema R.H. (2005). FoxM1 is required for execution of the mitotic programme and chromosome stability. Nat. Cell Biol..

[57] Costa R.H. (2005). FoxM1 dances with mitosis. Nat. Cell Biol..

[58] Wang X., Kiyokawa H., Dennewitz M.B., Costa R.H. (2002). The Forkhead Box m1b transcription factor is essential for hepatocyte DNA replication and mitosis during mouse liver regeneration. Proc. Natl. Acad. Sci. USA.

[59] Vigneron S., Sundermann L., Labbé J.C., Pintard L., Radulescu O., Castro A., Lorca T. (2018). Cyclin A-cdk1-dependent phosphorylation of bora is the triggering factor promoting mitotic entry. Dev. Cell.

[60] Pagliuca F.W., Collins M.O., Lichawska A., Zegerman P., Choudhary J.S., Pines J. (2011). Quantitative proteomics reveals the basis for the biochemical specificity of the cell-cycle machinery. Mol. Cell.

[61] Zhong Y., Yang J., Xu W.W., Wang Y., Zheng C.C., Li B., He Q.Y. (2017). KCTD12 promotes tumorigenesis by facilitating CDC25B/CDK1/Aurora A-dependent G2/M transition. Oncogene.

[62] Safi R., Nelson E.R., Chitneni S.K., Franz K.J., George D.J., Zalutsky M.R., Mcdonnell D.P. (2014). Copper signaling Axis as a target for prostate cancer therapeutics. Cancer Res..

[63] Jivan R., Damelin L.H., Birkhead M., Rousseau A.L., Veale R.B., Mavri-Damelin D. (2015). Disulfiram/copper-disulfiram damages multiple protein degradation and turnover pathways and cytotoxicity is enhanced by Metformin in oesophageal squamous cell carcinoma cell lines. J. Cell. Biochem..

[64] Palanimuthu D., Shinde S.V., Somasundaram K., Samuelson A.G. (2013). In vitro and in vivo anticancer activity of copper bis(thiosemicarbazone) complexes. J. Med. Chem..

[65] Huang X., Pierce L.J., Cobine P.A., Winge D.R., Spangrude G.J. (2009). Copper modulates the differentiation of mouse hematopoietic progenitor cells in culture. Cell Transplant..

[66] Jin Y., Zhang C., Xu H., Xue S., Wang Y., Hou Y., Kong Y., Xu Y. (2011). Combined effects of serum trace metals and polymorphisms of CYP1A1 or GSTM1 on non-small cell lung cancer: a hospital based case-control study in China. Cancer Epidemiol..

[67] Majumder S., Chatterjee S., Pal S., Biswas J., Efferth T., Choudhuri S.K. (2009). The role of copper in drug-resistant murine and human tumors. Biometals.

[68] Carpenter R.L., Gökmen-Polar Y. (2019). HSF1 as a cancer biomarker and therapeutic target. Curr. Cancer Drug Targets.

[69] Zhang H., Shao S., Zeng Y., Wang X., Qin Y., Ren Q., Xiang S., Wang Y., Xiao J., Sun Y. (2022). Reversible phase separation of HSF1 is required for an acute transcriptional response during heat shock. Nat. Cell Biol..

[70] Neef D.W., Jaeger A.M., Gomez-Pastor R., Willmund F., Frydman J., Thiele D.J. (2014). A direct regulatory interaction between chaperonin TRiC and stress-responsive transcription factor HSF1. Cell Rep..

[71] Kruta M., Sunshine M.J., Chua B.A., Fu Y., Chawla A., Dillingham C.H., Hidalgo San Jose L., De Jong B., Zhou F.J., Signer R.a.J. (2021). Hsf1 promotes hematopoietic stem cell fitness and proteostasis in response to ex vivo culture stress and aging. Cell Stem Cell.

[72] Li Y., Wang D., Ping X., Zhang Y., Zhang T., Wang L., Jin L., Zhao W., Guo M., Shen F. (2022). Local hyperthermia therapy induces browning of white fat and treats obesity. Cell.

[73] Jiang J.F., Zhou Z.Y., Liu Y.Z., Wu L., Nie B.B., Huang L., Zhang C. (2022). Role of Sp1 in atherosclerosis. Mol. Biol. Rep..

[74] Yuan S., Chen S., Xi Z., Liu Y. (2017). Copper-finger protein of Sp1: the molecular basis of copper sensing. Metallomics.

[75] Marin M., Karis A., Visser P., Grosveld F., Philipsen S. (1997). Transcription factor Sp1 is essential for early embryonic development but dispensable for cell growth and differentiation. cell.

[76] Ackerman C.M., Weber P.K., Xiao T., Thai B., Kuo T.J., Zhang E., Pett-Ridge J., Chang C.J. (2018). Multimodal LA-ICP-MS and nanoSIMS imaging enables copper mapping within photoreceptor megamitochondria in a zebrafish model of Menkes disease. Metallomics.

[77] Lorincz M.T. (2018). Wilson disease and related copper disorders. Handb. Clin. Neurol..

[78] Zhang P., He Q., Chen D., Liu W., Wang L., Zhang C., Ma D., Li W., Liu B., Liu F. (2015). G protein-coupled receptor 183 facilitates endothelial-to-hematopoietic transition via Notch1 inhibition. Cell Res..

[79] Liu J.X., Hu B., Wang Y., Gui J.F., Xiao W. (2009). Zebrafish eaf1 and eaf2/u19 mediate effective convergence and extension movements through the maintenance of wnt11 and wnt5 expression. J. Biol. Chem..

[80] Kossack M.E., Draper B.W. (2019). Genetic regulation of sex determination and maintenance in zebrafish (Danio rerio). Curr. Top. Dev. Biol..

[81] Liu J.X., Xu Q.H., Li S., Yu X., Liu W., Ouyang G., Zhang T., Chen L.L. (2017). Transcriptional factors Eaf1/2 inhibit endoderm and mesoderm formation via suppressing TGF-β signaling. Biochim. Biophys. Acta. Gene Regul. Mech..

[82] Zhang Y., Ding Z., Zhao G., Zhang T., Xu Q., Cui B., Liu J.X. (2018). Transcriptional responses and mechanisms of copper nanoparticle toxicology on zebrafish embryos. J. Hazard Mater..

[83] Soma S., Latimer A.J., Chun H., Vicary A.C., Timbalia S.A., Boulet A., Rahn J.J., Chan S.S.L., Leary S.C., Kim B.E. (2018). Elesclomol restores mitochondrial function in genetic models of copper deficiency. Proc. Natl. Acad. Sci. USA.

[84] Zhang C., Chen Y., Sun B., Wang L., Yang Y., Ma D., Lv J., Heng J., Ding Y., Xue Y. (2017). m(6)A modulates haematopoietic stem and progenitor cell specification. Nature.

[85] Liu J.X., Zhang D., Xie X., Ouyang G., Liu X., Sun Y., Xiao W. (2013). Eaf1 and Eaf2 negatively regulate canonical Wnt/beta-catenin signaling. Development.

[86] Pmd S. (2007).

