# Supplementary material for: Copper overload impairs hematopoietic stem and progenitor cell proliferation via prompting HSF1/SP1 aggregation and the subsequently downregulating FOXM1-Cytoskeleton axis
Source: iScience. 2023 Mar 14;26(4):106406. doi: 10.1016/j.isci.2023.106406 (PMC10050659; doi:10.1016/j.isci.2023.106406)
Supplement: Document S1. Figures S1–S11 and Schema 1 [file mmc1.pdf]

iScience, Volume *26*

Supplemental information

Copper overload impairs hematopoietic stem and progenitor cell proliferation *via* prompting HSF1/SP1 aggregation and the subsequently downregulating *FOXM1-Cytoskeleton* axis

LingYa Li, ZhiPeng Tai, WenYe Liu, Yi Luo, You Wu, ShuHui Lin, Mugen Liu, BaoXiang Gao, and Jing-Xia Liu

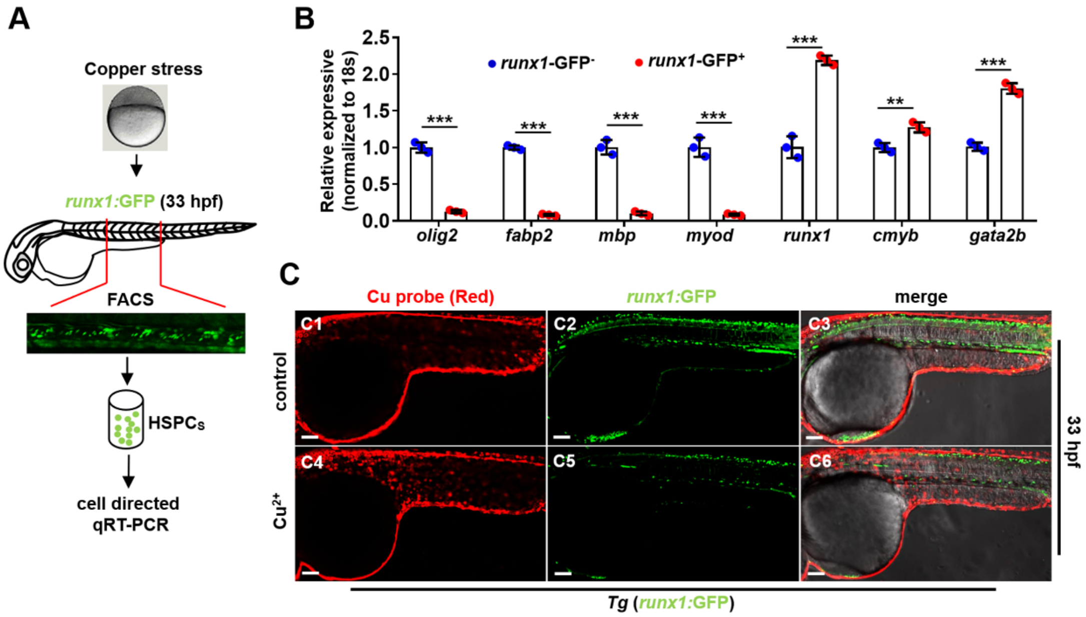

Figure S1

Figure S1. The HSPC identity of the *runx1*GFP^{+} cells and Cu ion probe staining in the whole embryos, Related to Figure 1.

(A) Schema for the experiments of (B).

(B) The collected *runx1*GFP^{+} cells exhibited abundant expression of HSPC markers *runx1*, *cmyb*, and *gata2a* while little expression of neural, liver, and muscle marker genes *oligo2*, *fabp2*, *mbp*, and *myod*, respectively.

(C) Distribution of Cu ion (Cu^{+} and Cu^{2+}) in Cu stressed *runx1*GFP^{+} embryos (labeled with Cu probe, Red^{+}).

C1-C6, lateral view, anterior to the left, and dorsal to the up. Data are mean ± SD. t-test, **P* < 0.05, ***P* < 0.01, ****P* < 0.001. Scale bars, 100 μm (C1-C6).

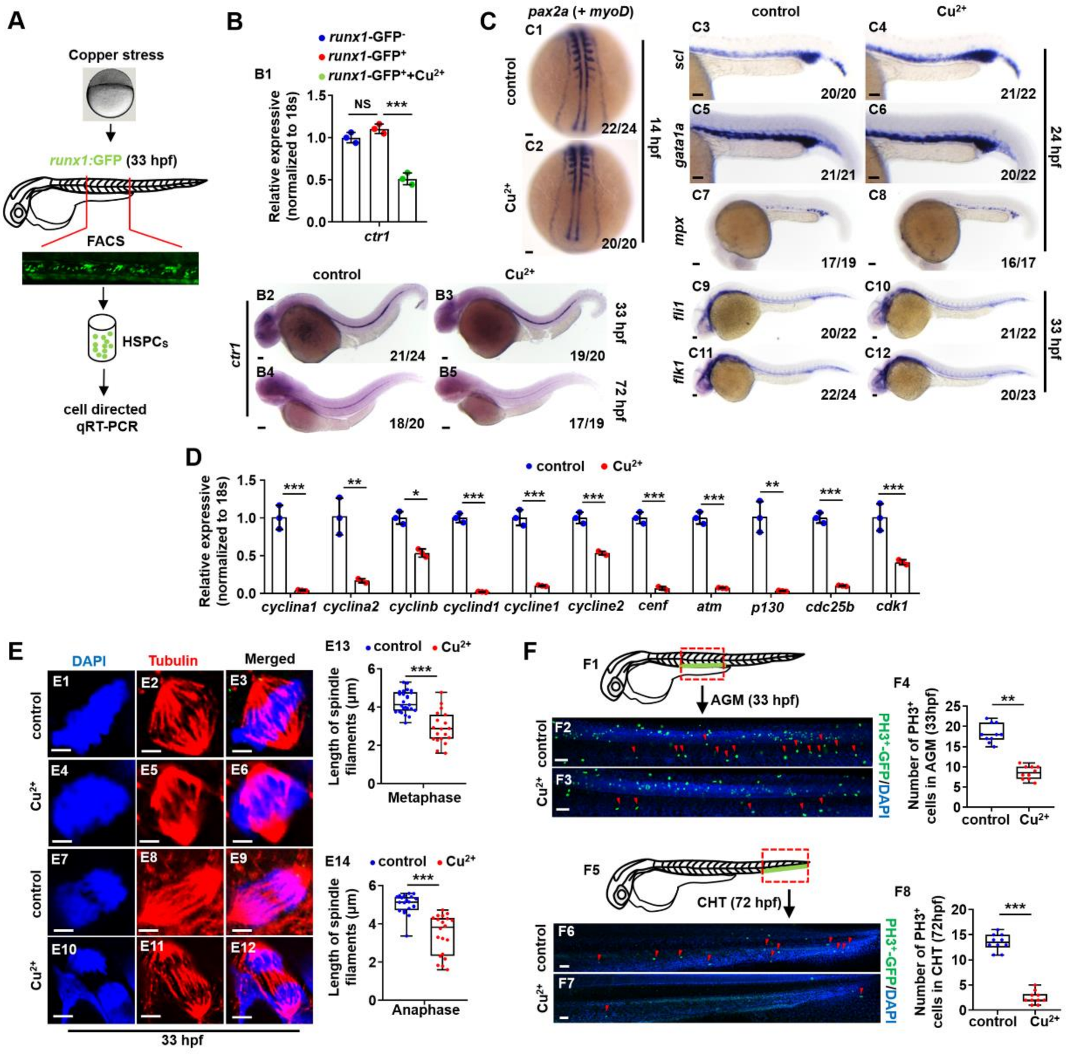

Figure S2

Figure S2. Cu overload impairs HSPC proliferation, Related to Figure 3.

(A) Schema for the experiments of (B1) and (D).

(B) Expression of Cu transporter gene *ctr1* in HSPCs at 33 hpf (B1) and the whole zebrafish embryos at 33 hpf and 72 hpf (B2-B5), respectively.

(C) Expression of *pax2a* and *myoD* genes in the posterior lateral mesoderm and trunk mesoderm respectively (C1, C2), and expression of *scl*, *gata1a*, *mpx* in zebrafish

embryos at 24 hpf (C3-C8) and vascular marker genes (*fli1*, *flk1*) in zebrafish embryos at 33 hpf (C9-C12) respectively.

(D) Expression of cell cycle regulator genes in HSPCs at 33 hpf.

(E) Mitotic malformation of non-*runx1*GFP$^+$ cells in AGM in Cu stressed embryos at 33 hpf. E1, E4, E7, E10, DAPI staining; E2, E5, E8, E11, anti-α-tubulin staining; E3, E6, E9, E12, merged. At least 10 mitotic non-*runx1*GFP$^+$ cells in more than 10 embryos were observed for each group. E13, E14, calculation of the length of spindles in metaphase non-*runx1*GFP$^+$ cells at 33 hpf and 72 hpf, respectively.

(F) PH3 immuno-staining analysis of HSPC proliferation in embryos at 33 hpf (F2, F3) and 72 hpf (F6, F7), respectively. F1, F5, AGM and CHT domain in embryos, respectively. F4, F8, calculation of HSPC proliferation in embryos from different groups.

C1, C2, dorsal view, anterior to the up. B2-B5, C3-C12, F2, F3, F6, F7, lateral view, anterior to the left, and dorsal to the up. Data are mean ± SD. t-test, $*P < 0.05$, $**P < 0.01$, $***P < 0.001$. Scale bars, 2 μm (E1-E12), 50 μm (B2-B5, C1-C12, F2, F3, F6, F7).

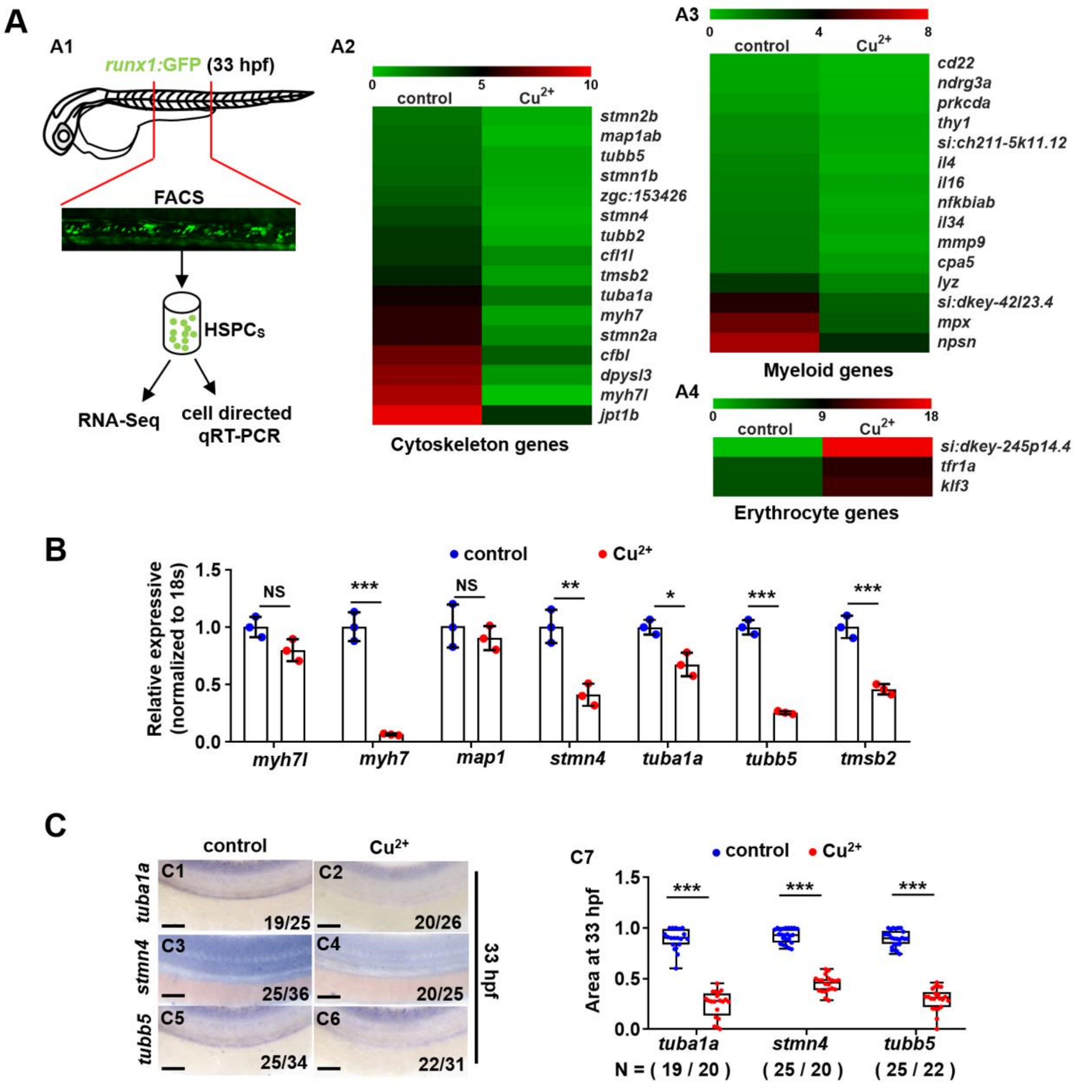

Figure S3

Figure S3. RNA-Seq for Cu stressed *runx1*GFP$^+$ cells and Cu overload destroyed the expression of cytoskeleton genes, Related to Figure 4.

(A) Schema (A1); (A2) Heatmap for down-regulated cytoskeleton genes in Cu stressed *runx1*GFP$^+$ cells at 33 hpf. (A3, A4) Heatmap for down-regulated myeloid cell homeostasis genes (A3) and up-regulated erythrocyte differentiation genes (A4) in Cu stressed *runx1*GFP$^+$ cells at 33 hpf.

(B) Expression of cytoskeleton genes in Cu stressed HSPCs at 33 hpf.

(C) WISH assays of the expression of cytoskeleton genes *tuba1a*, *stmn4*, and *tubb5* in the control and Cu stressed embryos at 33 hpf. C7, calculation of *tuba1a*, *stmn4*, and *tubb5* expression level in the Cu stressed and control embryos, respectively.

C1-C6, lateral view, anterior to the left, and dorsal to the up. Data are mean ± SD. t-test, *$P < 0.05$, **$P < 0.01$, ***$P < 0.001$. NS, not significant. Scale bars, 50 µm (C1-C6).

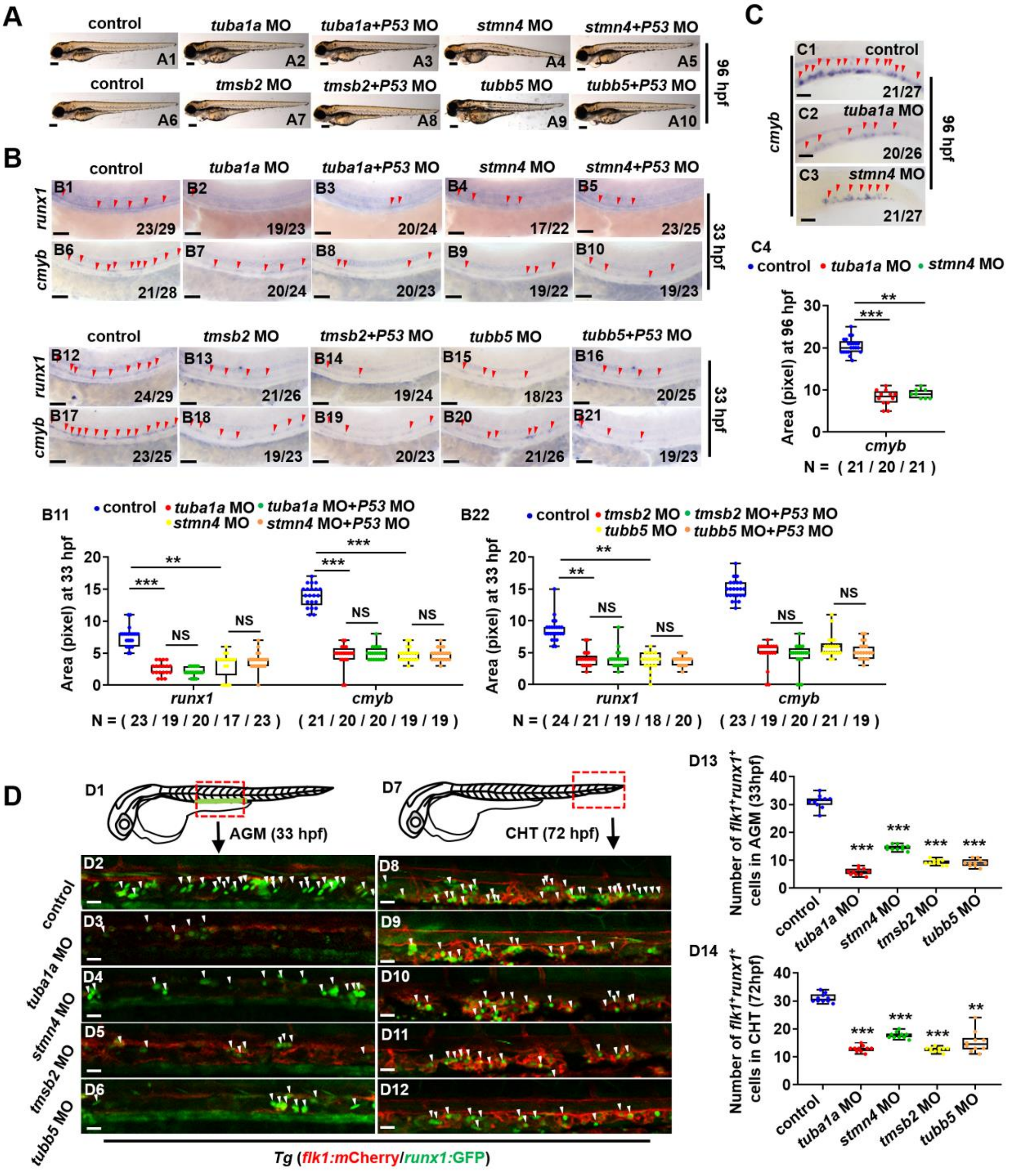

Figure S4

Figure. S4 Cytoskeleton morphants exhibited HSPC developmental defects, Related to Figure 4.

(A) The cytoskeleton gene morpholinos induced the zebrafish developmental defects at 96 hpf independent of *p53*.

(B) HSPC marker genes in *tuba1a*, *stmn4*, *tmsb4*, or *tubb5* morphants exhibited down-regulated expression at 33 hpf independent of *p53*. B11, B22, calculation of *runx1* and

cmyb expression in *tuba1a*, *stmn4*, *tmsb4*, or *tubb5* morphants, and for *tuba1a*, *stmn4*, *tmsb4*, or *tubb5* morphants co-injected with *p53* morpholino.

(C) HSPC marker genes exhibited down-regulated expression in *tuba1a*, *stmn4* morphants at 96 hpf. C4, calculation of *cmyb* expression in embryos from different groups.

(D) Knockdown of cytoskeleton genes *tuba1a*, *stmn4*, *tmsb4*, or *tubb5* induced significantly reduced expression of HSPCs in AGM (33 hpf) and CHT (72 hpf) domain in embryos. D13, D14, calculation of HSPC ($flk1^{+}runx1^{+}$ double positive at 33 hpf and $runx1^{+}$ at 72 hpf) cell number in *tuba1a*, *stmn4*, *tmsb4*, or *tubb5* morpholino injected embryos.

A1-A10, B1-B10, B12-B21, C1-C3, D2-D6, D8-D12, lateral view, anterior to the left, and dorsal to the up. Data are mean ± SD. t-test, *$P < 0.05$, **$P < 0.01$, ***$P < 0.001$. NS, not significant. Scale bars, 20 µm (D2-D6, D8-D12), 50 µm (B1-B10, B12-B21, C1-C3), and 100 µm (A1-A10).

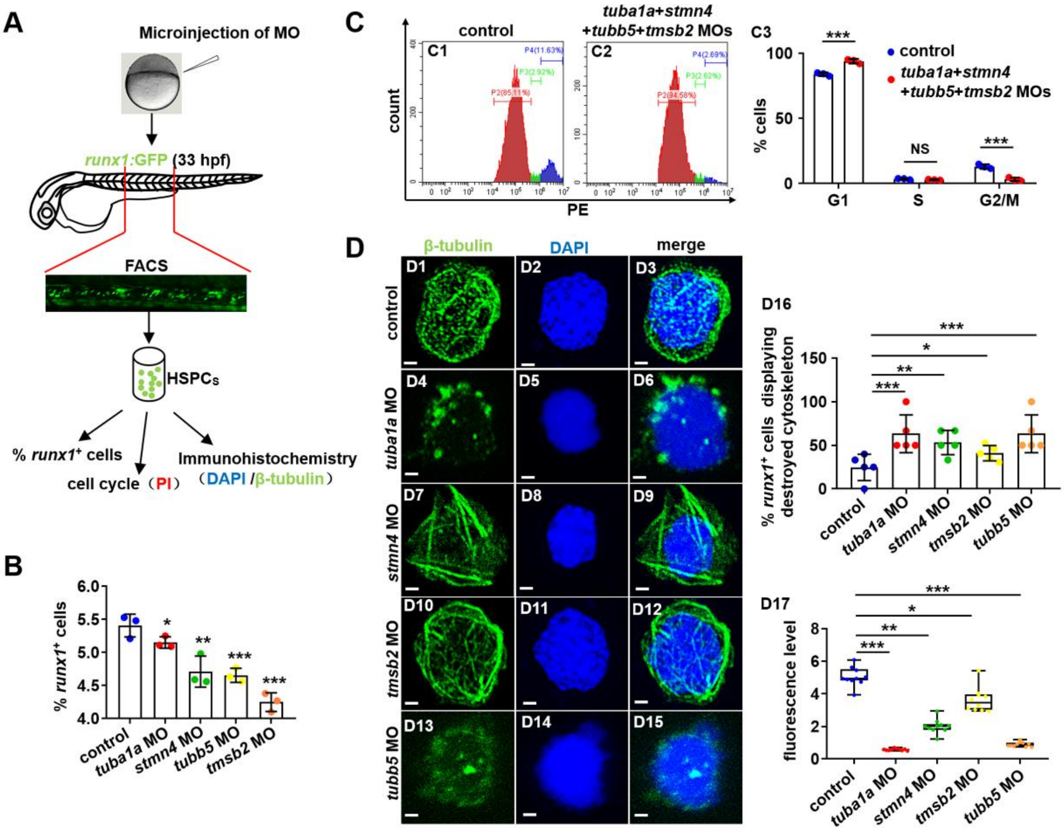

Figure S5

Figure S5. HSPCs from cytoskeleton morphants exhibited destroyed cell cycle and cytoskeleton protein distribution, Related to Figure 4.

(A) Schema for the experiments of (B), (C) and (D).

(B) The percentage of *runx1*GFP^{+} cells in different morphants at 33 hpf.

(C) Cell cycle of HSPCs in cytoskeleton gene knockdown (*tuba1a*-MO, *stmn4*-MO, *tubb5*-MO, and *tmsb2*-MO together) embryos at 33 hpf. C1, C2, flow cytometry (FACS) histogram; C3, cell cycle stage calculation.

(D) Cytoskeleton protein immune-fluorescence for HSPCs in *tuba1a*, *stmn4*, *tmsb2* or *tubb5* morphants at 33 hpf. D1, D4, D7, D10, D13, anti-β-tubulin staining; D2, D5, D8, D11, D14, DAPI staining; D3, D6, D9, D12, D15, merge. D16, the percentage of the *runx1*GFP^{+} cells exhibiting disrupted cytoskeleton in *tuba1a*, *stmn4*, *tmsb4* or *tubb5* morphants, respectively. D17, calculation of the fluorescence intensity in each cell. Data are mean ± SD. t-test, *$P < 0.05$, **$P < 0.01$, ***$P < 0.001$. NS, not significant. Scale bar, 1.2 μm (D1-D15).

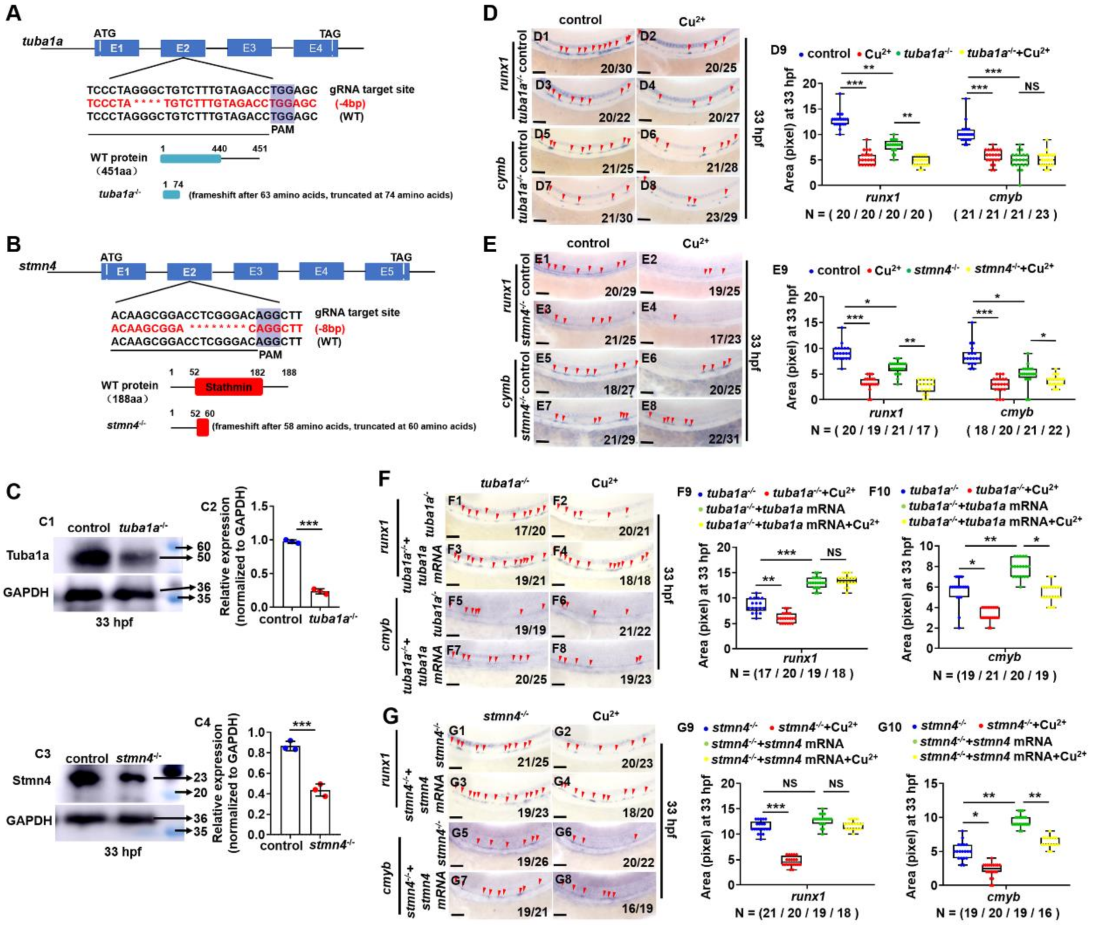

Figure S6

Figure S6. HSPC development in *tuba1a* and *stmn4* mutants, Related to Figure 4.

(A, B) Schematic diagrams showing the genomic structure and genetic mutations of zebrafish genes *tuba1a* (A) and *stmn4* (B).

(C) The protein level of Tuba1a (C1) and Stmn4 (C3) in *tuba1a* mutants and *stmn4* mutants at 33 hpf, respectively. GAPDH was used as an internal control.

(D) Expression of *runx1* and *cmyb* in *tuba1a* mutants with or without Cu stresses at 33 hpf. D9, calculation of *runx1* and *cmyb* expression in embryos from different groups.

(E) Expression of *runx1* and *cmyb* in *stmn4* mutants with or without Cu stresses at 33 hpf. E9, calculation of *runx1* and *cmyb* expression in embryos from different groups.
(F, G) Expression of *runx1* and *cmyb* in *tuba1a* or *stmn4* mutants with or without Cu stresses at 33 hpf; ectopic expression of *tuba1a* and *stmn4* mRNA could recover the expression of *runx1* and *cmyb* in Cu stressed *tuba1a* or *stmn4* mutants at 33 hpf. F9, F10, G9, G10, calculation of *runx1* and *cmyb* expression in embryos from different groups.
D1-D8, E1-E8, F1-F8, G1-G8, lateral view, anterior to the left, and dorsal to the up. Data are mean ± SD. t-test, $*P < 0.05$, $**P < 0.01$, $***P < 0.001$. NS, not significant. Scale bars, 50 μm (D1-D8, E1-E8, F1-F8, G1-G8).

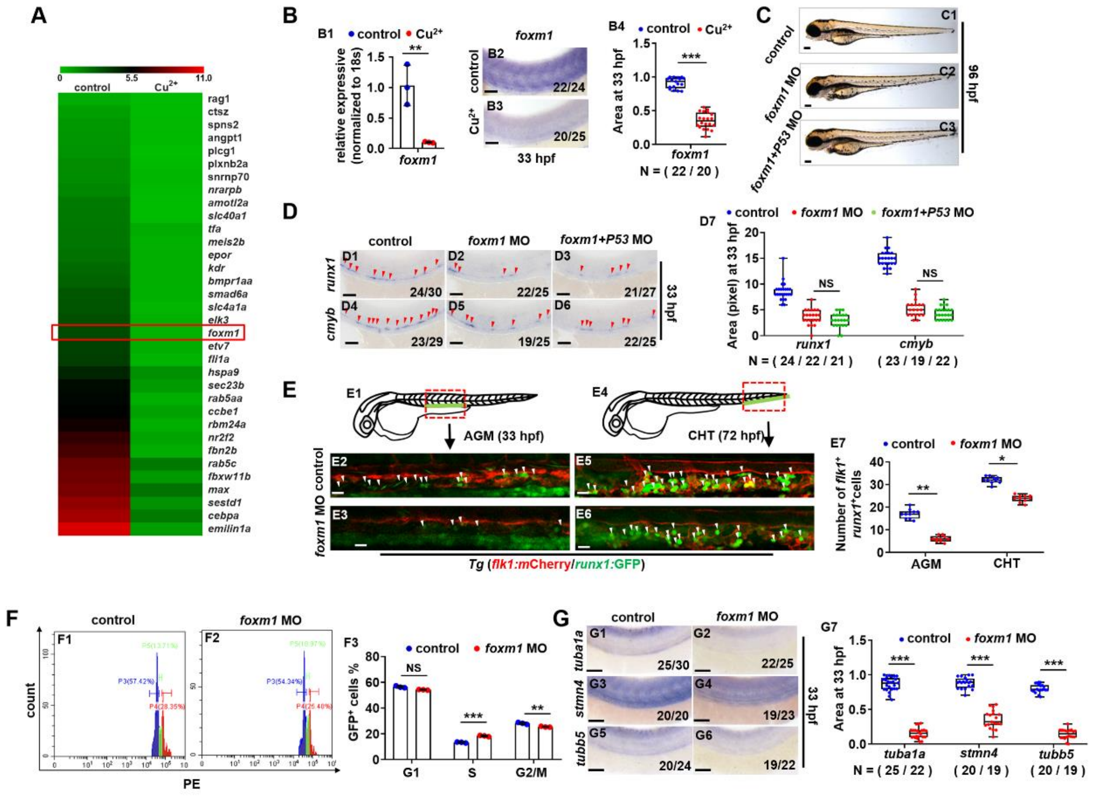

Figure S7

Figure S7. *Foxm1* mediates Cu overload induced HSPC proliferation blockage, Related to Figure 5.

(A) Heatmap for down-regulated genes such as *foxm1* in Cu stressed embyos at 33 hpf. (B) *Foxm1* exhibited significantly reduced expression in Cu stressed HSPCs (B1) and in AGM domain in Cu stressed embryos (B2, B3) at 33 hpf. B4, calculation of *foxm1* expression in embryos from different groups.

(C) Knockdown of *foxm1* induced the developmental defects at 33 hpf independent of *p53*

(D) HSPC marker genes in *foxm1* morphants exhibited down-regulated expression at 33 hpf independent of *p53*. D7, calculation of *runx1* and *cmyb* expression in *foxm1* morphant embryos, and *foxm1* morphants co-injected with *p53* morpholinos.

(E) Knockdown of *foxm1* induced significantly reduced HSPCs in AGM (33 hpf) and CHT (72 hpf) domain in embryos. E7, calculation of HSPC (*flk1*$^{+}$*runx1*$^{+}$ double positive at 33 hpf and *runx1*$^{+}$ at 72 hpf) cell number in *foxm1* morpholino injected embryos.

(F) Cell cycle of HSPCs in *foxm1* morphants at 33 hpf. F1, F2, flow cytometry (FACS) histogram; F3, cell cycle stage calculation.

(G) The expression of cytoskeleton genes *tubu1a*, *stmn4*, and *tubb5* in *foxm1* morphant embryos at 33 hpf. G7, calculation of *tubu1a*, *stmn4*, or *tubb5* expression in embryos from different groups.

B2, B3, C1-C3, D1-D6, E2, E3, E5, E6, G1-G6, lateral view, anterior to the left, and dorsal to the up. Data are mean ± SD. t-test, *$P < 0.05$, **$P < 0.01$, ***$P < 0.001$. NS, not significant. Scale bars, 20 µm (E2, E3, E5, E6), 50 µm (B2, B3, D1-D6, G1-G6) and 100 µm (C1-C3).

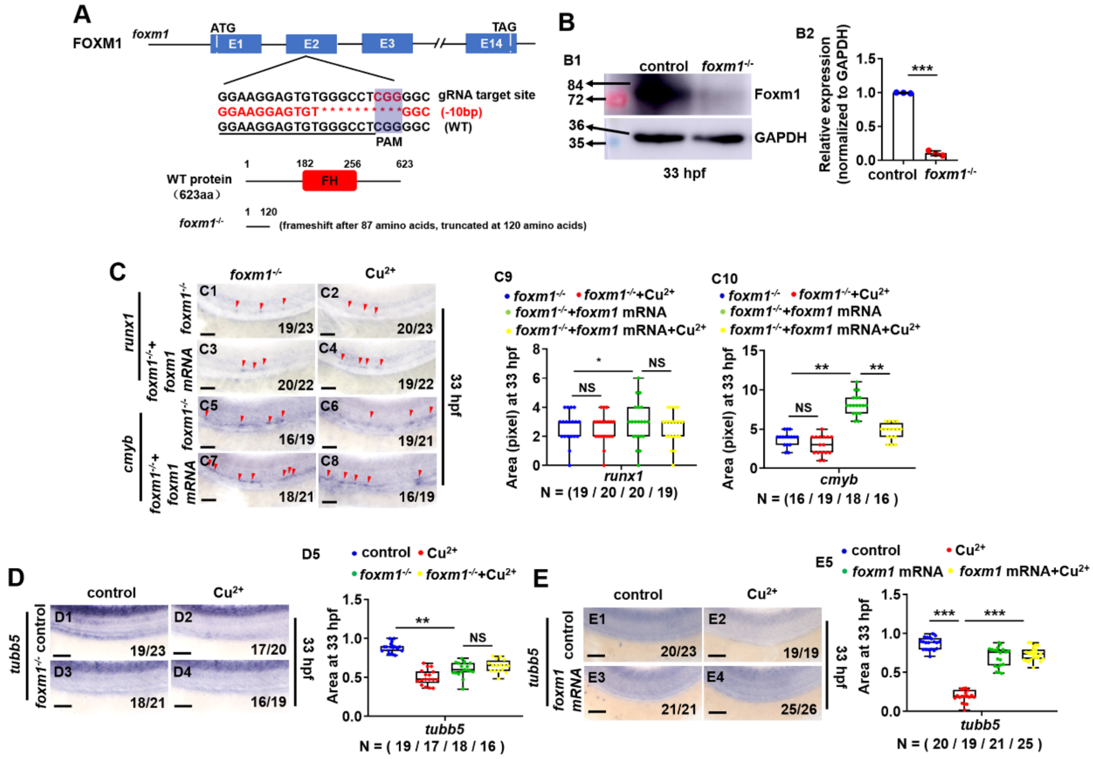

Figure S8

Figure S8. *Foxm1* functions up-stream of cytoskeleton genes in Cu overload induced HSPC proliferation blockage, Related to Figure 5.

(A) Schematic diagrams showing the genomic structure and genetic mutations of zebrafish gene *foxm1*.

(B) The protein level of Foxm1 in *foxm1* mutants with a 10-bp deletion at 33 hpf. GAPDH was used as an internal control.

(C) Expression of *runx1* and *cmyb* in *foxm1* mutants with or without Cu stresses at 33 hpf; ectopic expression of *foxm1* mRNA could recover the expression of *runx1* or *cmyb* in Cu stressed *foxm1* mutants at 33 hpf. C9, C10, calculation of *runx1* and *cmyb* expression in embryos from different groups.

(D) Expression of *tubb5* in *foxm1* mutants with or without Cu stresses at 33 hpf. D5, calculation of *tubb5* expression in embryos from different groups.

(E) Ectopic expression of *foxm1* mRNA could recover the expression of *tubb5* in Cu stressed embryos at 33 hpf. E5, calculation of *tubb5* expression in embryos from different groups.

C1-C8, D1-D4, E1-E4, lateral view, anterior to the left, and dorsal to the up. Data are mean ± SD. t-test, *$P < 0.05$, **$P < 0.01$, ***$P < 0.001$. NS, not significant. Scale bars, 50 μm (C1-C8, D1-D4, E1-E4).

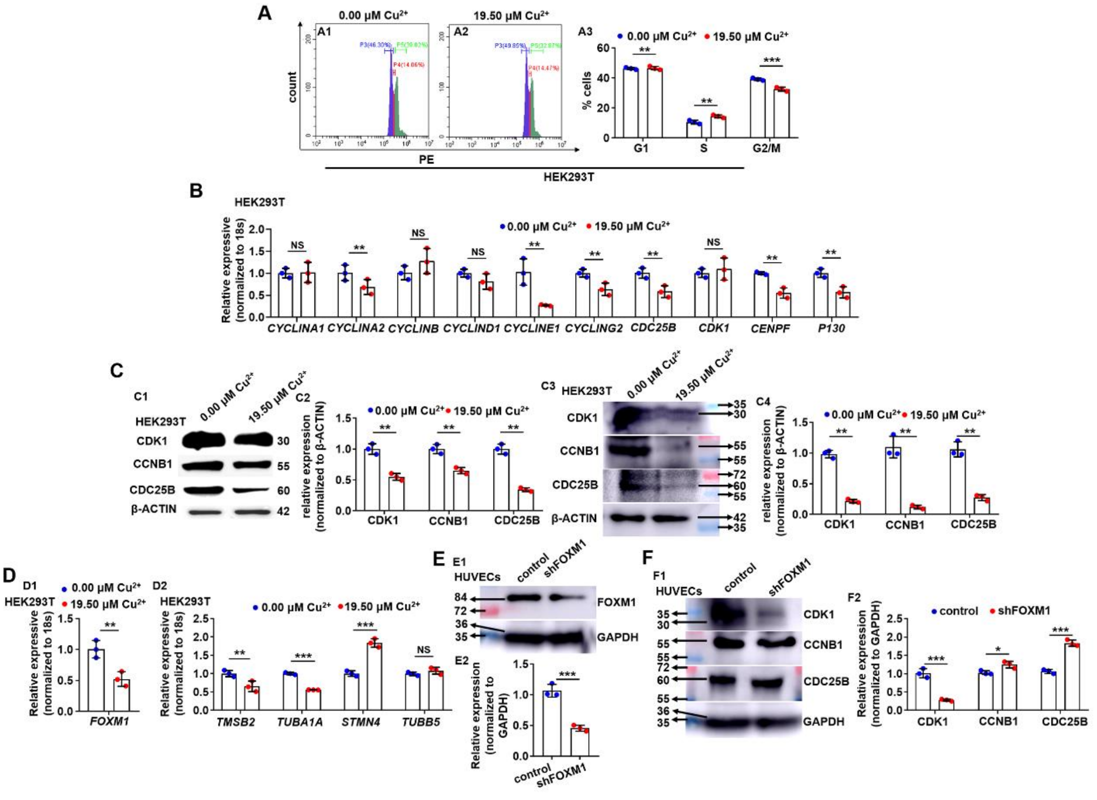

Figure S9

Figure S9. Cu overload impairs cell cycle in HEK293T cells, Related to Figure 7.

(A) Cell cycle in 19.50 μM Cu stressed HEK293T cells. A1, A2, flow cytometry (FACS) histogram; A3, calculation of cell cycle stage.

(B) Expression of cell cycle genes in Cu stressed HEK293T cells.

(C) Protein levels of cell cycle regulators CDK1, CCNB1, and CDC25B in Cu stressed HEK293T cells (C1, C3), β-ACTIN was used as an internal control.

(D) Expression of *FOXM1* genes (D1), and cytoskeleton genes (D2) in Cu stressed HEK293T cells.

(E) Protein level of FOXM1 was decreased in *FOXM1* knockdown (shFOXM1) HUVECs, GAPDH was used as an internal control.

(F) Protein levels of cell cycle regulators CDK1, CCNB1, and CDC25B in *FOXM1* knockdown (shFOXM1) HUVECs (F1), GAPDH was used as an internal control.

Data are mean ± SD. t-test, *$P < 0.05$, **$P < 0.01$, ***$P < 0.001$. NS, not significant.

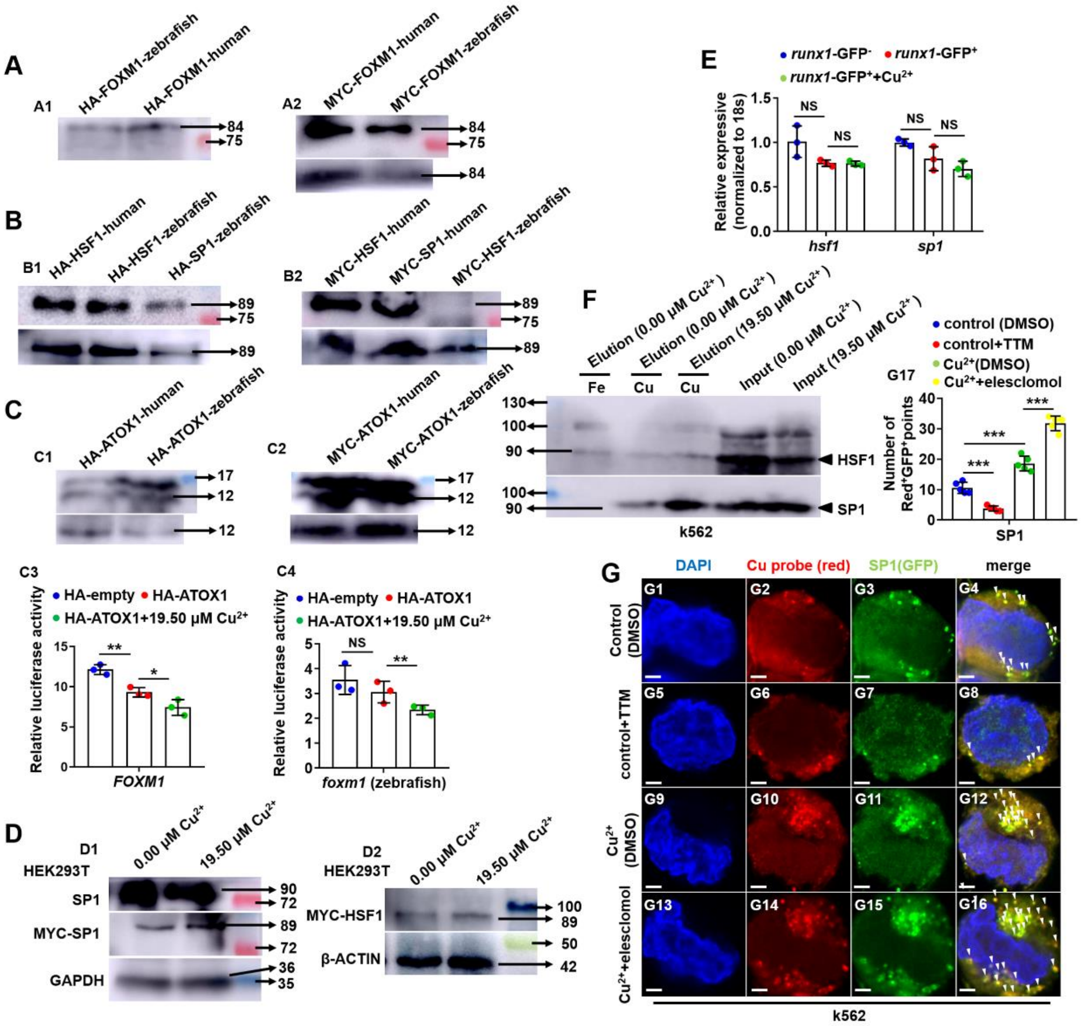

Figure S10

Figure S10. Cu overload impairs HSF1/SP1 transcriptional activities on *FOXM1*, Related to Figure 8.

(A, B, C) Ectopic expression of both zebrafish and human FOXM1 (A), HSF1 (B), SP1 (B) or ATOX1 (C1, C2), and their protein expression in HEK293T cell. Ectopic expression of zebrafish or human ATOX1 on transcriptional activities in human (C3) and zebrafish (C4) *FOXM1* promoters, respectively.

(D) Protein levels of SP1, MYC-SP1 (SP1), and MYC-HSF1 (HSF1) in Cu stressed mammalian cells.

(E) The expression of genes *sp1* and *hsf1* in *runx1*GFP^- cells, *runx1*GFP^+ cells and Cu stressed *runx1*GFP^+ cells, respectively.

(F) The binding of the indicated proteins (HSF1/SP1) to Cu^{2+} and Fe^{3+} was assessed by western blot analysis of eluted proteins from the indicated metal-loaded resins.

(G) Immunofluorescence imaging of Cu probe (Red^+) and GFP^+ (representing SP1 protein) double positive foci in cytoplasm of K562 cells. G17, calculation of the number of Red^+GFP^+ foci in K562 cells from different groups.

Data are mean ± SD. t-test, *$P < 0.05$, **$P < 0.01$, ***$P < 0.001$. NS, not significant. Scale bar, 2 μm (G1-G16).

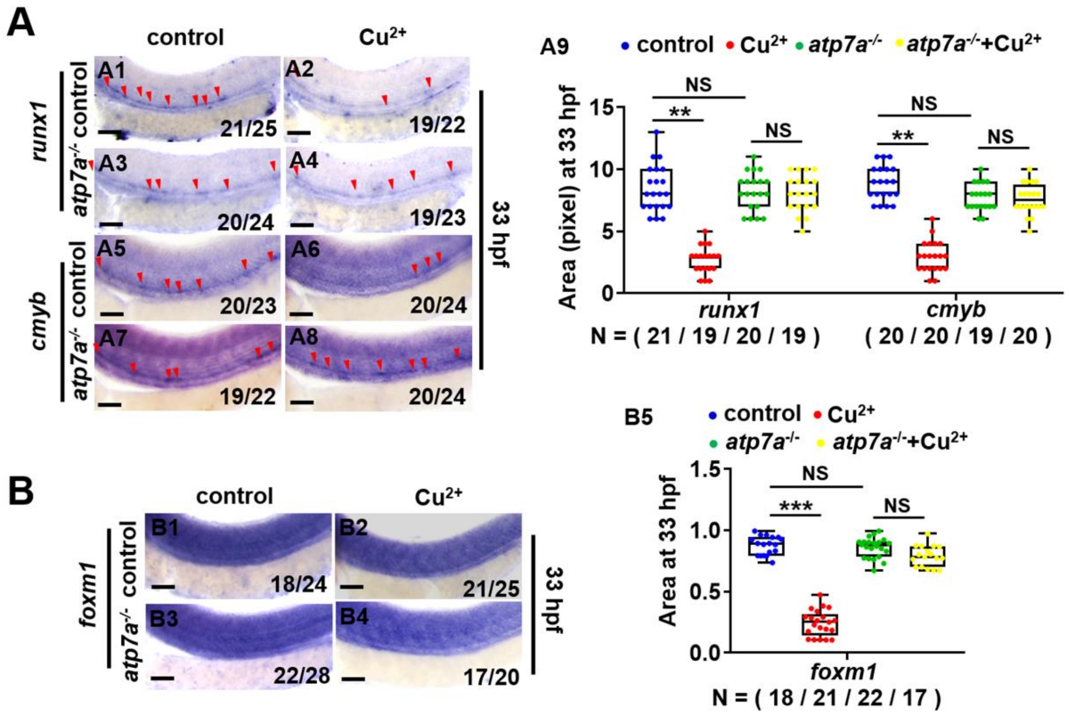

Figure S11

Figure S11. HSPC proliferation in genetic models with dysfunctional Cu homeostasis, Related to Figure 9.

(A) Expression of *runx1* and *cmyb* in *atp7a* mutants with or without Cu stresses at 33 hpf. A9, calculation of *runx1* and *cmyb* expression in embryos from different groups. (B) Expression of *foxm1* in *atp7a* mutants with or without Cu stresses at 33 hpf. B5, calculation of *foxm1* expression in embryos from different groups.

A1-A8, B1-B4, lateral view, anterior to the left, and dorsal to the up. Data are mean ± SD. t-test, **P* < 0.05, ***P* < 0.01, ****P* < 0.001. NS, not significant. Scale bar, 50 μm (A1-A8, B1-B4).

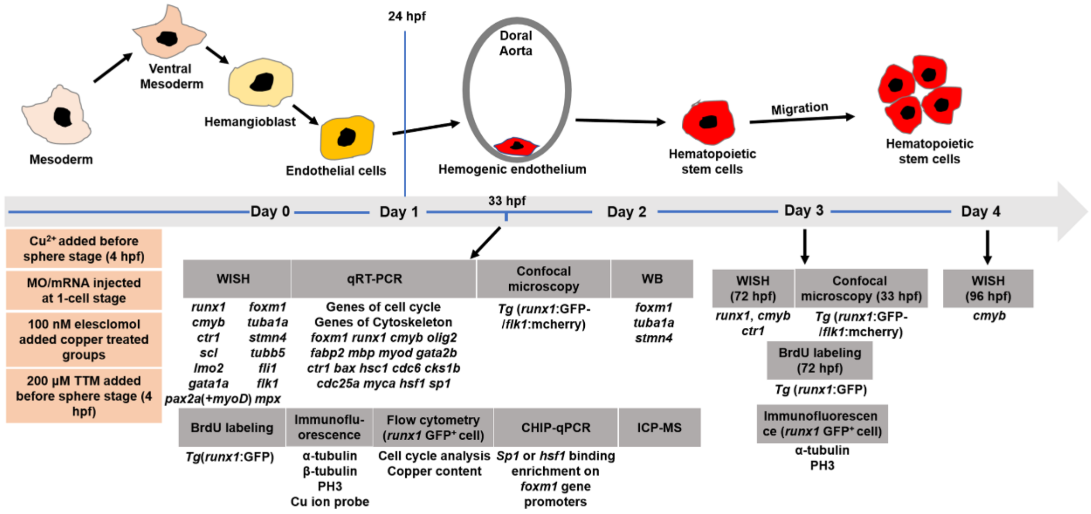

Schema 1

Schema 1. The embryos were collected at the indicated stages for experiments.
